# Evaluation of tree-based statistical learning methods for constructing genetic risk scores

**DOI:** 10.1186/s12859-022-04634-w

**Published:** 2022-03-21

**Authors:** Michael Lau, Claudia Wigmann, Sara Kress, Tamara Schikowski, Holger Schwender

**Affiliations:** 1 Mathematical Institute, Heinrich Heine University, Düsseldorf, Germany; 2 IUF – Leibniz Research Institute for Environmental Medicine, Düsseldorf, Germany

**Keywords:** Polygenic risk scores, Epistasis, Statistical learning, Random forests, Variable selection, Logic regression, Bagging, Elastic net, Simulation study

## Abstract

**Background:**

Genetic risk scores (GRS) summarize genetic features such as single nucleotide polymorphisms (SNPs) in a single statistic with respect to a given trait. So far, GRS are typically built using generalized linear models or regularized extensions. However, these linear methods are usually not able to incorporate gene-gene interactions or non-linear SNP-response relationships. Tree-based statistical learning methods such as random forests and logic regression may be an alternative to such regularized-regression-based methods and are investigated in this article. Moreover, we consider modifications of random forests and logic regression for the construction of GRS.

**Results:**

In an extensive simulation study and an application to a real data set from a German cohort study, we show that both tree-based approaches can outperform elastic net when constructing GRS for binary traits. Especially a modification of logic regression called logic bagging could induce comparatively high predictive power as measured by the area under the curve and the statistical power. Even when considering no epistatic interaction effects but only marginal genetic effects, the regularized regression method lead in most cases to inferior results.

**Conclusions:**

When constructing GRS, we recommend taking random forests and logic bagging into account, in particular, if it can be assumed that possibly unknown epistasis between SNPs is present. To develop the best possible prediction models, extensive joint hyperparameter optimizations should be conducted.

**Supplementary Information:**

The online version contains supplementary material available at 10.1186/s12859-022-04634-w.

## Background

The development of complex diseases depends on many factors such as genetic mutations, the lifestyle, or environmental factors. Investigating the effects of genetic variants across the human genome in genome-wide association studies (GWAS) has already revealed relevant risk base-pair alterations [1]. Single nucleotide polymorphisms (SNPs) may have only a very small effect on the investigated disease. However, when considered jointly, SNPs might be highly relevant [2, 3]. This behavior can be due to many independent SNPs exhibiting minor individual effects, or it can be caused by interactions of genetic variants, i.e., epistasis.

In consequence, summarizing relevant genetic effects in an individual while sufficiently predicting the risk for a certain disease, potentially jointly with non-genetic covariables, would be highly desirable. This would, on the one hand, allow to uncover underlying mechanisms related to this specific disease. On the other hand, accurately predicting the risk of disease for an individual could have a high impact on personalized medicine due to potentially being able to reduce the personal risk by taking specialized preventive measures if an individual has a high genetic risk for a certain disease [4, 5].

One promising approach for the assessment of an individual’s risk is the development of genetic risk scores (GRS). For the construction of GRS, one typically selects a subset of relevant SNPs from a biological pathway or a gene and calculates a weighted sum of the selected genetic variants.

Genome-wide approaches with a selection of genetic variants from across the whole genome resulting from prior knowledge are also possible for building GRS [6, 7]. However, such selections typically depend on large-scale association studies in which single SNPs were tested individually with regard to the phenotype. Thus, interacting variants which do not exhibit substantial marginal effects might be left out although SNP level interactions might contribute to disease risk [8, 9]. In this context, an alternative to conventional GWAS for identifying disease-related SNPs might be genome-wide association interaction studies (GWAIS) [9].

The standard procedure for the computation of the GRS is the usage of external weights [10, 11], ideally determined from independent association studies such as GWAS or GWAIS. However, there might be no appropriate association study for the regarded outcome or population available such that suitable weights have to be gathered in a different way.

Internal GRS weights can be estimated by regarding the problem of constructing GRS as a supervised statistical learning problem, where the response would be the disease status or a quantitative biological variable such as the glucose level. In this case, the predictors are genetic variants of the specific pathway or gene, where SNPs are usually coded by the number of minor alleles for this individual. The estimation of proper weights or fitted models which generalize well, i.e., which represent the whole population reasonably well and not only the available sample, requires the partitioning of the whole data set into training and test data sets. Dudbridge [3] and Hüls et al. [11] found in their studies that a random close to one-half split generalizes well. Sufficient samples are necessary in the test data set for evaluating the association of the GRS with the response which especially holds true for gene-environment interaction (GxE) studies in which more parameters are to be estimated. A GxE interaction is present if, for different genotypes, different disease susceptibilities to an environmental factor are underlying, e.g., if an individual has a high genetic risk for a certain disease which is enabled by an environmental factor [12].

So far mainly linear methods such as generalized linear models (GLM) or regularization methods based on GLMs, such as the lasso [13] or one of its generalizations, the elastic net [14], have been used in the construction of GRS [11, 15, 16]. The elastic net offers the advantage of properly handling highly correlated predictors, e.g., SNPs in linkage disequilibrium (LD), by employing an $L_2$ regularization while performing a variable selection due to the $L_1$ regularization. Nonetheless, these regularized linear regression methods cannot directly take interactions between predictors into account (unless specific interaction terms were specified prior to applying them) and the assumption of an additive relationship between the response and the input variables has to be fulfilled. Therefore, the usage of algorithms which are able to develop more general models and which in fact can find and take interesting interactions into account might be preferable.

The tree-based statistical learning method random forests [17] is well-known and widely used among a variety of use cases [e.g., [18–20]]. It builds several individual classification or regression trees (CART) [21], which are fitted by a non-linear recursive partitioning algorithm, and combines them to one strong ensemble. For a low to moderate amount of SNPs ($< 100$), it has been shown that the classic random forests algorithm is able to properly uncover SNP interactions even when the corresponding marginal effects are negligible [22].

Another tree-based non-linear statistical learning procedure is logic regression [23] which mainly considers binary predictors. It searches for Boolean expressions of the input variables and combines multiple expressions in a GLM and already has been used in applications to SNP data [24–26]. Both tree-based methods are theoretically able to cover each possible prediction scenario for categorical input data. However, their model fitting techniques are highly different.

To the best of our knowledge, it has barely been investigated yet whether the aforementioned statistical learning algorithms can be used as alternative procedures to conventional GRS construction approaches. For random forests, some publications suggest that the ensemble method is able to outperform conventional linear methods such as logistic regression, odds ratio scores or the lasso [27, 28]. However, more recent studies which considered genome-wide risk scores, i.e., GRS constructed using SNPs from all over the genome and not just single genes or pathways, were not able to verify that random forests should be used over linear approaches [29, 30]. In the context of disease risk prediction, e.g., Yoo et al. [31] regarded random forests, logic regression, and logistic regression without penalization in one simple gene-gene interaction simulation study and additionally in a real data application. In their analyses, the tree-based algorithms could induce higher predictive performances than logistic regression. Nonetheless, multi-faceted analyses taking different realistic data scenarios into account are necessary in order to draw meaningful conclusions about the appropriateness of the tree-based methods for the construction of GRS.

The classic random forests and logic regression algorithms have some shortcomings. In particular, random forests can severely overfit the data [32] and logic regression can lead to highly variant models [24]. Thus, we additionally considered modifications of the classic algorithms to overcome these drawbacks.

In this article, we, therefore, evaluate random forests, logic regression, and extensions of these methods in an extensive simulation study and an application to a real data set from a German cohort study for the construction of GRS and compare the results to the elastic net.

## Methods

### Construction of genetic risk scores

Let ${\mathcal {D}}_{\mathrm {train}} = \lbrace ({\varvec{x}}_i, y_i) \rbrace _{i=1}^N$ be a training data set with *N* observations and binary outcomes $y_i \in \lbrace 0, 1 \rbrace$. Further assume that each input vector ${\varvec{x}}_i$ is a collection of *p* biallelic SNPs, i.e., taking values in the *p*-dimensional space $\lbrace 0,1,2 \rbrace ^p$, where 0 codes the homozygous reference, 1 the heterozygous variant, and 2 the homozygous variant. Then the problem of constructing a GRS model consists of fitting a proper function

$$\begin{aligned} {\mathrm {GRS}}: \lbrace 0,1,2 \rbrace ^p \rightarrow [ 0, 1 ]. \end{aligned}$$

The target space is equal to the probability scale [0, 1], since ${\mathrm {GRS}}({\varvec{x}})$ should be an estimate of ${\mathbb {P}}(Y = 1 \mid {\varvec{X}} = {\varvec{x}})$, i.e., the probability of being a case given some SNPs ${\varvec{x}}$. This fitting procedure is conducted on a designated training data set. Independent observations from a test data set ${\mathcal {D}}_{\mathrm {test}}$ are used to evaluate the GRS, i.e., ${\mathrm {GRS}}({\varvec{x}})$ for $({\varvec{x}}, \cdot ) \in {\mathcal {D}}_{\mathrm {test}}$.

**Fig. 1:**
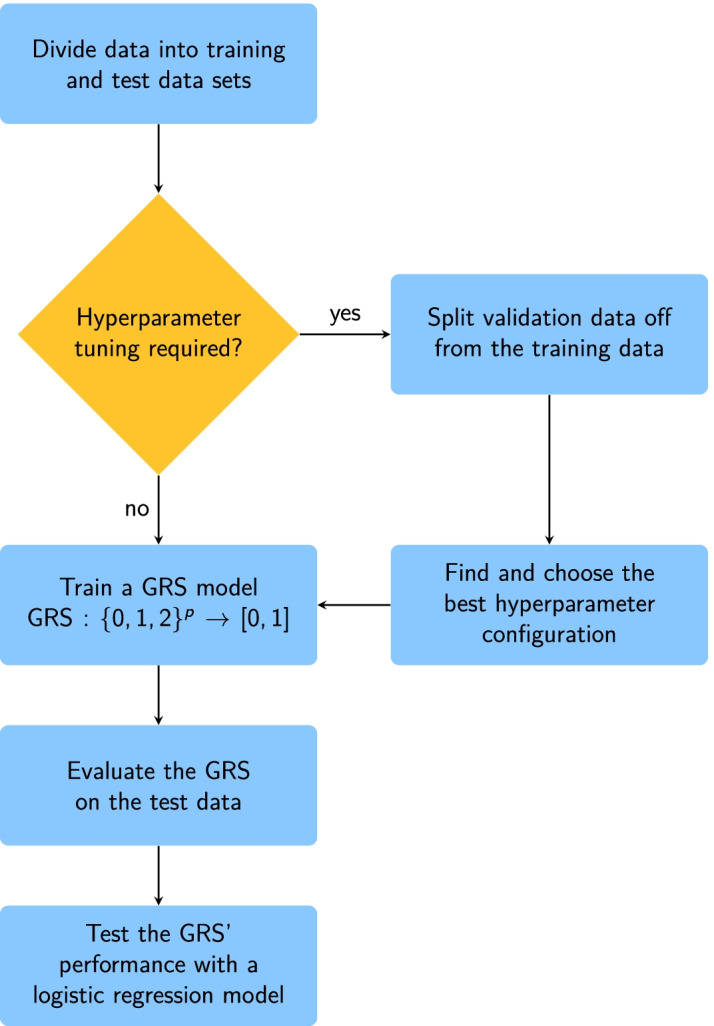
Workflow of constructing and evaluating genetic risk scores

An overview of the workflow for fitting and evaluating GRS models using the statistical learning approach is given in Fig. 1.

### Random forests

**Fig. 2:**
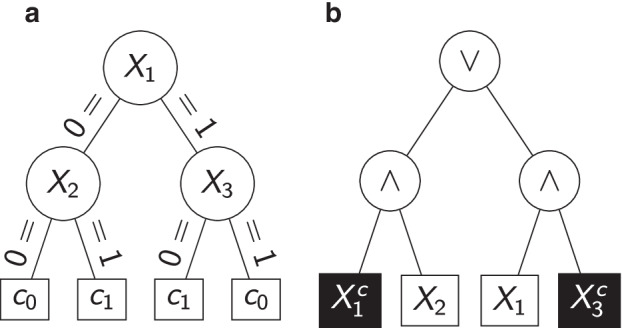
Exemplary tree models for three binary input variables $X_1$, $X_2$ and $X_3$ predicting two different classes $c_0$ and $c_1$. In **a**, a classification tree is shown. **b** depicts a logic tree describing the Boolean expression $(X_1^c \wedge X_2) \vee (X_1 \wedge X_3^c)$. Here, a true Boolean expression is identified as class $c_1$ and $c_0$ otherwise. Negated input variables/leaves are marked by white letters on a black background. Both trees are equivalent, i.e., they perform the same predictions for each predictor setting

In random forests, multiple classification or regression trees (CART) [21] with injected randomness are built to form one strong ensemble. From a graph-theoretical point of view, decision trees are usually binary trees in which each inner knot represents a split based on a predictor and each leaf (terminal node) describes a prediction scenario. Figure 2a illustrates an exemplary classification tree with four disjoint prediction scenarios. New predictions start at the root node and follow the respective edge until a leaf is reached.

Decision trees are induced by a recursive greedy splitting algorithm which searches at each inner node for the best possible split with respect to an impurity measure. The impurity measure is a quantifier for the homogeneity of respective nodes. For binary classification trees, the Gini impurity

$$\begin{aligned} i(t) = 2 \cdot P(Y = 1 \mid {\varvec{X}} \in t) P(Y = 0 \mid {\varvec{X}} \in t) \end{aligned}$$

for empirical probabilities $P(Y = c \mid {\varvec{X}} \in t)$ that the response *Y* is equal to class *c* given that the input vector ${\varvec{X}}$ falls into the regarded node *t* is usually chosen.

The tree induction procedure can be locally terminated by stopping criteria. When a node should not be split, it is declared as a leaf and has to receive a prediction value. For classification trees, this is usually the class with the highest empirical probability in the regarded branch.

However, single decision trees suffer from the instability problem which states that a small noise-like modification of the training data set may lead to a disproportional modification of the fitted tree. This issue is mainly caused by the greedy fashion of choosing splits [33].

Random forests tries to address this issue. The algorithm employs bagging [34] which draws a bootstrap sample of the available data for each individual tree as its training data set. The tree fitting procedure is further randomized by adjusting the splitting algorithm to choose $mtry \le p$ predictors from the total set of input variables at every inner node which qualify for the best split. $mtry$ is a hyperparameter usually chosen as $\sqrt{p}$ or *p*/3 which should be properly tuned in certain applications. Based on these randomizations, the resulting model averages the individual trees, i.e., for classification trees, the class which is classified most often will be chosen as the prediction.

### Random forests for constructing genetic risk scores

If one is aiming at constructing GRS for binary traits, one has to keep in mind that probability estimates for showing the regarded feature are needed instead of class estimates. Random forests based on classification trees can be used for probability estimation by averaging the number of trees which voted for class 1 [35]. However, if we, e.g., assume that the true risk for being a case would be equal to $80\%$ and that all classification trees properly recognize this fact and, therefore, predict class 1 for this particular setting, the forest risk estimate would be given by $100\%$. Thus, for this reason, we consider probability estimation trees [36] which hold risk estimates in their leaves in contrast to classifications. These estimates are usually chosen as the empirical branch probabilities from which classification trees also draw their estimates. Random forests based on probability estimation trees average the probability estimates of the individual trees similar to regression trees.

If SNP variables coded as 0, 1, or 2 are interpreted as quantitative variables, decision trees and random forests are able to split with respect to $(\lbrace 0 \rbrace , \lbrace 1, 2 \rbrace )$ or $(\lbrace 0, 1 \rbrace , \lbrace 2 \rbrace )$, thus, considering both dominant and recessive modes of inheritance. Therefore, SNPs are directly used as input variables when employing random forests.

### Random forests VIM

One issue that arose when fitting the first GRS models with random forests in our initial experiments was a substantial overfitting which could be observed by comparing the test and training data errors. Therefore, performing an appropriate variable selection prior to fitting the final random forests models might reduce the overfitting and lead to better results for noise-intensive data. Kursa and Rudnicki [37] proposed an iterative variable selection approach which relies on variable importance measures (VIM) and which they called Boruta. The permutation VIM can be calculated using the out-of-bag observations for each tree, thus, avoiding an overfitting of the VIM itself. In each iteration, the Boruta approach adds for each predictor variable a shadow variable with the same values but randomly permutes them to destroy a potential predictor-response relationship for this variable. Next, a random forest on this extended set of input variables is fitted and the evaluated VIMs for these shadow variables are used to approximate the distribution of VIMs for non-influential input variables. The computed VIMs of the original variables are then compared to the VIMs of the shadow variables in statistical tests for importance. In particular, the maximum observed importance of all shadow variables is used to decide whether an original variable is temporarily classified as important. More specifically, if a variable yields an importance higher than the maximum observed importance among all shadow variables, it will be temporarily marked as important. Several iterations of creating shadow variables, fitting random forests, and computing VIMs are used to perform binomial tests, which regard how often the variable was temporarily marked as important, testing the alternative of greater or smaller VIM realizations, i.e., important or unimportant variables. More precisely, these binomial tests are based on the null hypothesis that the probability of the regarded input variable yielding a higher VIM than the maximum VIM of all shadow variables is equal to 0.5. The significance threshold of the binomial tests is set to 1%, which is also the recommended threshold by the authors of the Boruta approach. Compared to other random-forest-based variable selection methods such as the Vita algorithm proposed by Janitza et al. [38] which relies on negative VIM values, the Boruta approach does not require a vast amount of (noninfluential) input variables.

As an alternative procedure, we also tried the variable selection method by Altmann et al. [39], which relies on random permutations of the response variable. However, in our experiments, the Boruta approach yielded more stable results in general. In particular, even when considering different significance thresholds for the approach by Altmann et al. [39], the Boruta procedure still could induce more stable variable selections, i.e., leading to variable selections that did not severely differ between independent replicates. This observation is in line with the analyses by Degenhardt et al. [40] who provide an in-depth comparison of various random forests variable selection methods.

Hence, we fitted ordinary random forests with probability estimation trees and random forests based on the Boruta variable selection which we call random forests VIM in the following. For random forests, we used the R package ranger [41]. For random forests VIM, the R package Boruta [37], that also relies on the ranger package, was used.

### Logic regression

Logic regression [23] is a tree-based statistical learning algorithm which is specifically tailored to binary input variables. It searches for ideal Boolean expressions of those and works with binary tree representations of Boolean expressions, logic trees. Logic trees hold the Boolean operators $\wedge$ (AND) or $\vee$ (OR) in their inner nodes and contain predictor variables or their negations (indicated through $^c$) in their terminal nodes. Figure 2b depicts an exemplary logic tree which is equivalent to the exemplary classification tree from Fig. 2a, i.e., both trees perform the same predictions for each realization of the three input variables. The interpretation as a Boolean expression is obtained recursively by combining expressions in a bottom-up fashion, yielding $(X_1^c \wedge X_2) \vee (X_1 \wedge X_3^c)$ for the logic tree from Fig. 2b.

Logic trees themselves can only be used for binary classification tasks, since they represent logic expressions so that their output is also either 0 or 1. To generalize their usage for, e.g., risk prediction, Ruczinski et al. [23] proposed using logic trees $L_1, \ldots , L_M$ as predictors in a GLM

$$\begin{aligned} g({\mathbb {E}}[Y \mid {\varvec{X}} = {\varvec{x}}]) = \beta _0 + \beta _1 L_1({\varvec{x}}) + \ldots + \beta _M L_M({\varvec{x}}) \end{aligned}$$

considering an appropriate link function *g* such as the ${\mathrm {logit}}$ function ${\mathrm{logit}}(p) = \log (p/(1-p))$ for a binary response.

The total model fitting procedure consists of finding the most appropriate logic tree(s). In practice, for each model, a set of neighbor states is defined by simple adjustments of the current model. The moves used in logic regression consist of exchanging variables and operators, adding or removing branches, splitting or removing variables, and adding or removing trees. This set of moves ensures that from every state, every other possible state can be reached in a final number of steps. For more details, see [23].

Based upon this methodology, two model search algorithms are used in practice:

- a greedy search which evaluates each neighbor of a given state and moves to the best one
- simulated annealing [42], a stochastic search algorithm which only considers one random neighbor per iteration and can also move to worse states to prevent being stuck in a local minimum.

Model ranking is performed using a score function which is chosen to be the deviance for the logistic model. The model which yields the best score among all models visited in the search is chosen as the resulting model. Irrespective of using the greedy approach or simulated annealing, one should configure the model size hyperparameters, i.e., the total number of trees and the total number of leaves, to obtain the best fit on the entire population. For fitting conventional logic regression models, we used the R package LogicReg [43] and used simulated annealing as the search procedure.

### Logic regression for constructing genetic risk scores

SNP variables coded as 0, 1, or 2 can be biologically meaningful divided into two binary variables, in ${\mathrm {SNP}}_D = {\mathbbm {1}}({\mathrm {SNP}} \ne 0)$, coding for a dominant effect, and in ${\mathrm {SNP}}_R = {\mathbbm {1}}({\mathrm {SNP}} = 2)$, coding for a recessive effect. With these two binary variables, interactions can be properly expressed. For example, consider a scenario where two SNPs influence the disease risk in such a way that the risk is significantly increased if and only if for both SNPs their respective minor allele occurs at least once. With Boolean logic, this can be expressed as ${\mathrm {SNP}}_{1,D} \wedge {\mathrm {SNP}}_{2,D}$. It might also be possible that two risk-increasing SNPs with a dominant mode of inheritance can only elevate the disease risk once, i.e., if both statuses occur, the risk is not increased beyond the first elevation. This scenario can also be expressed with Boolean logic as ${\mathrm {SNP}}_{1,D} \vee {\mathrm {SNP}}_{2,D}$. Furthermore, SNPs in high linkage disequilibrium (LD) that are, therefore, highly correlated can also be properly addressed with the logical OR. One LD block might then be expressed as a chain of OR-concatenated SNPs, a disjunction. Thus, for the construction of GRS with logic regression, each SNP is divided into two binary variables prior to applying the procedure.

### Logic bagging

As an alternative to an exhaustive search with simulated annealing, we also considered applying bagging [34] to logic regression models fitted with a greedy search. We call this approach logic bagging. In contrast to conventional logic regression, logic bagging fits ensembles of individual logic regression models and, similar to random forests, predictions are made using the average of the predictions of the individual logic regression models. This approach is still computationally expensive when using an adequate amount of bagging iterations (e.g., 500) but reduces the variance and does not require the tuning of a cooling schedule. Logic bagging is implemented in the R package logicFS [44]. For fitting logic bagging models, the greedy search is employed mainly due to computational reasons. In particular, in Additional file 1: Fig. S1, the model fitting times are depicted. For example, for fitting and evaluating a single logic bagging model consisting of 500 logic regression models fitted via simulated annealing, it would take about $500 \cdot 28.82s \approx 4h$ using the mean model fitting and evaluation time of 28.82*s* for logic regression.

### Elastic net

The elastic net [14] is a regularized linear regression model which combines

- the lasso (least absolute shrinkage and selection operator) [13], i.e., $L_1$ regularized regression that reduces the estimate of the regression coefficients of non-influential predictors to zero, therefore, excluding non-informative input variables,
- and ridge regression [45], i.e., $L_2$ regularized regression for properly handling highly correlated predictors by assigning similar weights to such predictors.

Elastic net, hence, uses a penalty term given by

$$\begin{aligned} R_{\alpha } (\varvec{\beta }) := \frac{1}{2} (1-\alpha ) ||\varvec{\beta }||_2^2 + \alpha ||\varvec{\beta }||_1 \end{aligned}$$

for the regression coefficients $$\varvec{\beta } = \begin{pmatrix} \beta _1&\ldots&\beta _p \end{pmatrix}^T$$ in the fitting procedure solving the optimization problem

1 $$\begin{aligned} \min _{\beta _0, \varvec{\beta }} \left\{ -\frac{1}{N} \ell (\beta _0, \varvec{\beta }) + \lambda R_\alpha (\varvec{\beta }) \right\} \end{aligned}$$

for the log-likelihood function $\ell$. In this article, binary outcomes are considered. Thus, the logistic regression approach for elastic net was employed.

Here, $\lambda \ge 0$ determines the strength of the regularization, i.e., for larger values of $\lambda$, the penalty $\lambda R_\alpha (\varvec{\beta })$ increases, thus, favoring coefficient vectors with smaller norms, i.e., more loosely fitting models. The parameter $\alpha \in [0,1]$ configures the balance between the lasso and ridge regression, i.e., for $\alpha = 0$, one would perform ordinary ridge regression and for $\alpha = 1$, one would apply the lasso. Therefore, these two hyperparameters have to be tuned properly.

In practice, the model coefficients are estimated by employing coordinate descent as optimization algorithm to solve the minimization problem (1) and taking advantage of the fact that similar values of $\lambda$ lead to similar model coefficients for a fast fitting of different $\lambda$ settings [46]. We used the R package glmnet [46] with cross-validation for fitting elastic net models.

The common procedure when constructing GRS with regularized regression procedures such as elastic net is to use the $\lbrace 0,1,2 \rbrace$ coding for each SNP in the model [11, 16]. We, therefore, follow in our comparison this standard procedure and use the $\lbrace 0,1,2 \rbrace$ coding in the elastic net.

If interaction effects between SNPs should be included in the elastic net model, they have to be explicitly specified prior to fitting the model. However, in practice, it is usually unknown which loci might interact. Including all possible interactions between SNPs becomes rapidly infeasible, as for a moderate amount of SNPs, the number of possible interaction terms might already be too high. For example, for 50 SNPs, there exist more than $10^{15}$ interaction terms. The standard procedure for constructing GRS with linear methods such as the elastic net is to only consider marginal genetic effects [16]. Thus, we follow in our evaluations this common procedure and do not include interactions in the elastic net models.

## Simulation studies

The tree-based statistical learning methods random forests, random forests VIM, logic regression, and logic bagging were evaluated and compared to the elastic net in a simulation study considering three scenarios with several different settings. All SNPs were drawn independently resembling LD-based pruned or clumped SNPs. All simulations and analyses were performed with R version 4.0.3 [47]. Data sets for all simulation scenarios were generated using the R function simulateSNPglm from the scrime package [48].

### General workflow

The general workflow for generating the data sets for the simulation study is given as follows for each of the simulation settings, which are described in detail afterwards.

1. Choose the fixed data parameters, i.e., the odds ratios, number of SNPs, sample size and simulation design.
2. For each SNP, draw a random minor allele frequency (MAF).
3. Randomly generate the genotypes based on the MAFs.
4. If continuous covariables are to be included, randomly generate the data for these variables.
5. Randomly generate the outcome according to the linear predictor.
6. Evaluate the fraction of cases in the generated outcome and tune the prevalence such that this fraction becomes approximately balanced, i.e., yielding $\sim 50\%$ cases. This involves going back to step 5.
7. Create 100 independent data sets for a certain setting using the steps 2–5 for each repetition.

### Simulation setups

#### Marginal genetic effects

In a first step, we focused on main effects, which represents the ideal case for the elastic net, since no interactions are considered here and the individual effects behave additively with each other. Similar to Hüls et al. [49], we considered six SNPs influencing the value of the outcome, where we simulated a dominant effect for each of these SNPs. Thus, we first considered data sets generated from a logistic regression model

2 $$\begin{aligned} {\mathrm {logit}}({\mathbb {P}}(Y = 1)) = \beta _0 + \sum _{i=1}^6 \beta _i \cdot {\mathbbm {1}}({\mathrm {SNP}}_i \ne 0) = \beta _0 + \sum _{i=1}^6 \beta _i \cdot {\mathrm {SNP}}_{i,D}. \end{aligned}$$

In order to draw conclusions for different realistic scenarios, we varied three parameters:

- the effect size, i.e., the odds ratio, of each influential SNP which can be configured by specifying $\exp (\beta _i)$ [50],
- the intensity of statistical noise which we adjusted by adding non-influential SNPs to each data set,
- and the sample size of each data set.

To achieve nearly case-control study-like designs, we configured the prevalence, i.e., $\left( 1+\exp (-\beta _0)\right) ^{-1}$ [50], to result in nearly balanced data sets for each regarded odds ratio. The MAF was drawn randomly for each SNP and for each data set from the interval [0.15, 0.45] similar to Pan et al. [51]. For each scenario, we generated 100 independent data sets, i.e., we performed 100 replications. Table 1 lists the regarded settings for the aforementioned simulation parameters.

**Table 1:** Parameter settings for the first simulation scenario resulting in 27 settings in total

Parameter	Considered realizations
Odds ratio	1.2, 1.5, 1.8
Amount of noise SNPs	4, 14, 44
Sample size	500, 1000, 2000
Prevalence	Resulting in balanced data sets
MAF	Randomly chosen from [0.15, 0.45]
Repetitions	100

#### Dominant interactions of SNPs

In a second simulation scenario, we additionally considered a gene-gene interaction, i.e., an interaction between SNPs. More specifically, we here always considered three SNPs with low main effects, i.e., odds ratios of 1.2 and a dominant mode of inheritance, since we focused on marginal effects in the first scenario. Additionally, we included an interaction term between two SNPs whose odds ratio was varied. Similar to the first scenario, we also varied the amount of statistical noise, i.e., the number of SNPs for which no effect on the outcome is intended. Furthermore, we considered three sub designs that determine which SNPs interact. The data was generated following models such as

3 $$\begin{aligned} \begin{aligned} {\mathrm {logit}}({\mathbb {P}}(Y = 1)) = \beta _0 + \sum _{i=1}^3 \beta _i \cdot {\mathrm {SNP}}_{i,D} + \beta _4 \cdot {\mathrm {SNP}}_{j,D} \cdot {\mathrm {SNP}}_{k,D}. \end{aligned} \end{aligned}$$

The indices $(j, k) \in \lbrace (1,2), (1,4), (4,5) \rbrace$ determine whether both interacting SNPs also do have marginal effects, only one of them exhibits a main effect, or if they only are influential when considered jointly. The prevalence was again configured by $\beta _0$ to approximately achieve case-control-balanced study designs. The MAF was randomly chosen in the interval [0.15, 0.45] and the sample size was fixed to 2000 observations per data set, since we only considered weak marginal effects. 100 independent data sets for each setting were analyzed using a cyclic scheme such as in the first simulation scenario. The study parameters for the second simulation scenario are summarized in Table 2.

**Table 2:** Study parameters for the second simulation scenario resulting in 45 settings in total

Parameter	Considered realizations
Odds ratio of gene-gene interaction	1.2, 1.5, 1.8, 2.1, 2.4
Amount of noise SNPs	5, 15, 45
Interacting SNPs (j, k)	(1, 2), (1, 4), (4, 5)
Sample size	2000
Prevalence	Resulting in balanced data sets
MAF	Randomly chosen from [0.15, 0.45]
Repetitions	100

#### Gene-environment interactions

In the final simulation scenario, we added two correlated continuous variables to the true underlying model from which one forms a GxE interaction with a SNP. One of these two variables exhibits a marginal effect on the outcome, while the second variable only influences the outcome if a certain risk allele occurs at least once. The data for this scenario was generated considering the model

4 $$\begin{aligned} \begin{aligned} {\mathrm {logit}}({\mathbb {P}}(Y = 1))&= \beta _0 + \sum _{i=1}^3 \beta _i \cdot {\mathrm {SNP}}_{i,D} + \beta _4 \cdot {\mathrm {SNP}}_{1,D} \cdot {\mathrm {SNP}}_{4,D} \\&\quad + \beta _5 \cdot E_1 + \beta _6 \cdot E_2 \cdot {\mathrm {SNP}}_{j,D}. \end{aligned} \end{aligned}$$

Similar to the gene-gene interaction simulation scenario, the effects for the first three SNPs were fixed to odds ratio of 1.2, 1.5, and 1.8, respectively. The interaction between ${\mathrm {SNP}}_1$ and ${\mathrm {SNP}}_4$ received a fixed odds ratio of 1.8, since in this analysis, the focus lies on the GxE interaction. The index $j \in \lbrace 2,5 \rbrace$ determines whether the SNP in the GxE interaction also exhibits a moderate marginal effect or if this SNP only influences the outcome in interaction with the continuous variable $E_2$. The odds ratios of the terms involving the continuous variables $E_1$ or $E_2$ were specified per IQR (interquartile range) of the respective environmental variable as it is regularly done when performing analyses of GxE interactions [11]. For the continuous variable $E_1$, the (marginal) odds ratio was fixed to 1.2 per IQR. The odds ratio of the GxE interaction between ${\mathrm {SNP}}_j$ and $E_2$ was varied between 1.2 and 2.4. The continuous variables were generated following a multivariate normal distribution, i.e.,

$$\begin{aligned} \begin{pmatrix} E_1 \\ E_2 \end{pmatrix}\ \sim \ {\mathcal {N}}_2\left( \begin{pmatrix} \mu \\ \mu \end{pmatrix},\ \sigma ^2 \begin{pmatrix} 1 &{} \rho \\ \rho &{} 1 \end{pmatrix} \right) . \end{aligned}$$

In particular, the mean $\mu$ was set to 20, the variance $\sigma ^2$ was chosen as 10 and the correlation $\rho$ between these two variables was chosen as either 0.5 or 0.9, resembling moderately and highly correlated variables, respectively. The prevalence was again configured by $\beta _0$ to approximately achieve case-control-balanced study designs. The MAF was randomly chosen in the interval [0.15, 0.45] and the sample size was fixed to 2000 observations per data set as in the previous simulation scenario. The number of additional noise SNPs was fixed to 45. 100 independent data sets for each setting were analyzed. The study parameters for the third simulation scenario are summarized in Table 3. In GxE interaction studies, GRS are usually constructed only using the available genetic data [11]. Thus, we constructed the GRS without utilizing the environmental variables.

**Table 3:** Study parameters for the third simulation scenario resulting in 20 settings in total

Parameter	Considered realizations
Odds ratio of GxE interaction	1.2, 1.5, 1.8, 2.1, 2.4
Amount of noise SNPs	45
Interacting GxE SNP j	2, 5
Correlation between $E_1$ and $E_2$	0.5, 0.9
Sample size	2000
Prevalence	Resulting in balanced data sets
MAF	Randomly chosen from [0.15, 0.45]
Repetitions	100

### Analysis of association and predictive strength

To evaluate and compare the different statistical learning methods in their ability to construct GRS, a cyclic training-validation-test data set scheme was considered. In the *i*-th repetition of this cyclic scheme, the *i*-th data set ${\mathcal {D}}_i$, $i \in \lbrace 1, \ldots , 100 \rbrace$, was used to train the GRS with the different statistical learning methods. For the evaluation of the performance of these methods, the succeeding data set ${\mathcal {D}}_{i+1}$ if $i \ne 100$ and ${\mathcal {D}}_1$ otherwise was chosen to be the independent test data set. For tuning the hyperparameters (see “Section Hyperparameter optimization”), we chose the preceding data set, i.e., ${\mathcal {D}}_{i-1}$ if $i \ne 1$ and ${\mathcal {D}}_{100}$ otherwise as validation data.

Since all data sets were generated independently, the cyclic scheme is equivalent to a conventional training-validation-test data set approach in which each of the 100 data sets is once used as training set, once as test set, and once as validation set in a cyclic manner. Due to the high computational costs when considering many different parameter configurations, hyperparameter tuning was performed by averaging the performances over the first 10 validation iterations for each simulation setting and each parameter setting. The setting which yielded the highest validation AUC across the average over these 10 repetitions was chosen as the fixed setting for the particular simulation setting.

The standard approach for testing the association considers the GRS as a predictor in a conventional regression model [2]. For binary outcomes, the logistic regression model is fitted on the test data. The logistic regression model maps the linear predictor with the logistic function from $(-\infty , +\infty )$ to (0, 1). Thus, the GRS (probability estimates) are transformed to the scale of the linear predictor by applying the inverse of the logistic function, the ${\mathrm {logit}}$ function. In summary, the univariate association model

5 $$\begin{aligned} {\mathrm {logit}}({\mathbb {P}}(Y = 1 \mid {\mathrm {GRS}})) = \beta _0 + \beta _1 \cdot {\mathrm {GRS}} \end{aligned}$$

is constructed using

$$\begin{aligned} \Big \{ \left( {\mathrm {GRS}}({\varvec{x}}), y\right) := \left( {\mathrm {logit}}\left( {\mathrm {GRS}}_\mathrm {raw}({\varvec{x}})\right) , y \right) \;\big |\ ({\varvec{x}}, y) \in {\mathcal {D}}_\mathrm {test} \Big \} \end{aligned}$$

for raw risk predictions of the fitted GRS model ${\mathrm {GRS}}_\mathrm {raw}$.

For statistically assessing this association, we conducted Wald tests testing the alternative that the GRS is associated with the response. Based on these test results, we estimated the statistical power and the type I error rate for analyzing and comparing the ability of properly recognizing signals in the genetic data by the GRS construction procedures. The statistical power, which is given by the probability that the GRS is correctly recognized as influential on the response, can be estimated by the fraction of logistic models with statistically significant predictors under all cases which rely on theoretically influential genetic data. The type I error rate, i.e., the false positive rate, can be estimated by the fraction of significantly recognized GRS under all cases in which the response and the predictors are actually independent.

To compare the predictive strength of GRS, which is probably most relevant, we calculated the area under the curve (AUC) with respect to the receiver operating characteristic (ROC). This metric offers two main advantages over classification measures such as the accuracy, sensitivity, or specificity. First, it does not depend on the classification threshold which perhaps should be tuned. Second, the AUC can handle imbalanced data sets due to simultaneously regarding sensitivity and specificity. Moreover, the AUC has an intuitive interpretation as the probability that a random observation from the entire population of cases yields a higher risk estimate than a randomly chosen control from the population [52].

Additionally, we evaluated the classical classification metrics accuracy, sensitivity, and specificity. In particular, we performed hard classifications on the resulting logistic regression model containing the GRS using a classification threshold of 0.5, i.e., classifying an observation as a case if it is predicted that the probability of being a case is higher than the probability of being a control and classifying an observation as a control otherwise. Using these classifications, the overall accuracy, sensitivity, and specificity as defined, e.g., in Alberg et al. [53] were evaluated. The accuracy was not explicitly adjusted for the prevalence, since we generated approximately case-control-balanced data sets in the simulation study, thus, yielding a prevalence of 50%. However, the main purpose of GRS does not lie in hard classifying observations as cases or controls. Instead, GRS are used for estimating individual risks, e.g., in precision medicine or for uncovering biological mechanisms involved in the development of diseases. Therefore, a metric such as the AUC which simultaneously considers different sensitivities and specificities seems to be preferable in the evaluation of the performance of GRS.

### Hyperparameter optimization

Certain statistical learning procedures require the optimization of hyperparameters using independent validation data sets. This also holds true for the algorithms considered in this article. Table 4 lists the regarded hyperparameter configurations, where each possible combination of these parameters has to be considered in the parameter tuning. A description of each of these parameters is given in Additional file 1: Section 2. For random forests, we fixed the number of total trees grown to 2000, which is a sufficiently large number of trees in our applications, since in preliminary experiments, we could observe that the validation AUC converged using smaller amounts of trees. Analogously, we fixed the number of bagging iterations for logic bagging to 500. The cooling schedule in logic regression was configured manually by observing the cooling behavior for different settings and choosing a start temperature and end temperature such that around 90% of the proposed models were accepted at the beginning of the algorithm and close to no models were accepted when approaching the end temperature. The amount of simulated annealing iterations was fixed to 500000. The regularization parameter $\lambda$ for the elastic net was automatically chosen by employing cross-validation in the respective fitting processes and selecting the value which minimizes the loss.

For each considered statistical learning method, a more detailed workflow for tuning and training the respective models is depicted in Additional file 1: Section 3.

**Table 4:** Regarded hyperparameter settings

Algorithm	Hyperparameter	Considered realizations
Random forests & random forests VIM	mtry	$$\left\lfloor \begin{pmatrix} 0.5&1&2 \end{pmatrix} \cdot \lfloor \sqrt{p} \rfloor \right\rfloor$$
	min.node.size	$$\left\lfloor \begin{pmatrix} 0.01&0.05&0.1 \end{pmatrix} \cdot N \right\rfloor$$
	num.trees	2000
Logic regression & logic bagging	ntrees	$$\begin{pmatrix} 1&2&3&4&5&6 \end{pmatrix}$$
	nleaves	$$\begin{pmatrix} 1&2&\ldots&9&10 \end{pmatrix}$$ (Simulation studies)
		$$\begin{pmatrix} 1&2&\ldots&19&20 \end{pmatrix}$$ (Real data application)
Logic regression	Cooling schedule	Experimental
	Simulated annealing iterations	500000
Logic bagging	Bagging iterations	500
Elastic net	$\alpha$	$$\begin{pmatrix} 0.5&0.75&0.9&0.99 \end{pmatrix}$$
	$\lambda$	Cross-validation

The mentioned hyperparameter names are the names of the corresponding arguments in the respective software packages. For a description of the parameters, see Additional file 1: Section 2

### Results of the simulation studies

#### Marginal genetic effects

**Fig. 3:**
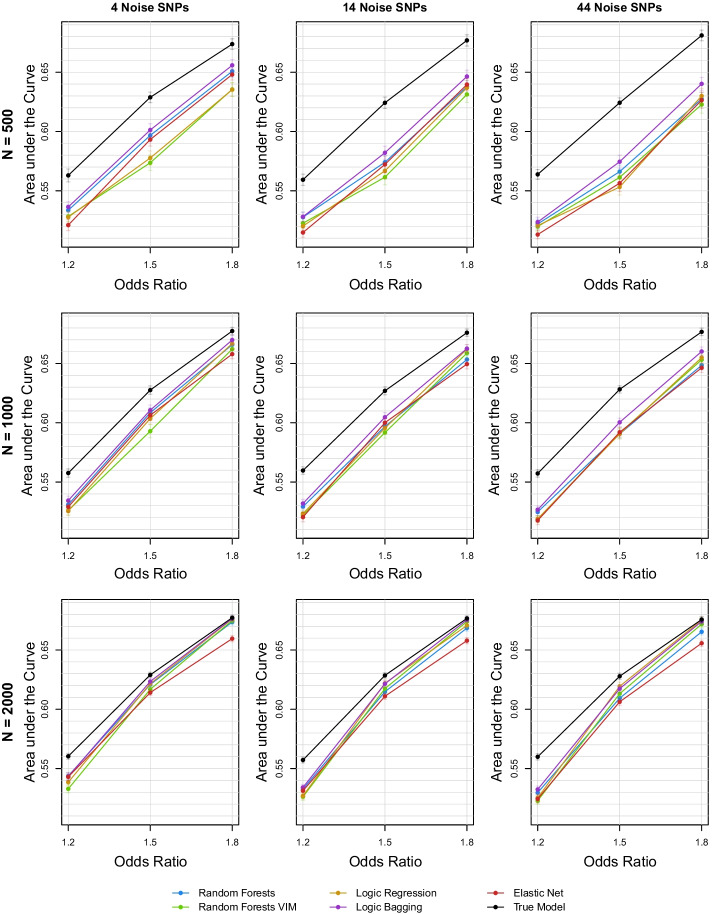
Mean AUC for random forests, random forests VIM, logic regression, logic bagging, elastic net, and the true underlying model in the first simulation scenario considering marginal effective SNPs evaluated on the test data

Figure 3 summarizes the AUC for each of the 27 regarded settings in the main effects simulation scenario. In Additional file 1: Fig. S2, corresponding asymptotic 95% confidence intervals are depicted. Most notably, logic bagging leads in almost every scenario to the highest AUC. For strong effects and large data sets, ordinary logic regression induces similar or even better results which are comparable to the true underlying model. Especially for weak effects, ordinary random forests yields comparably high values for the AUC. Unsurprisingly, random forests with a prior variable selection is more effective in relation to the other procedures when considering a higher amount of statistical noise. For less noisy data, random forests VIM cannot compete with the other tree-based methods and shows high variations. The elastic net yields inferior results for large data sets and large effect sizes and also has difficulties detecting a signal for the more challenging scenarios, i.e., for small odds ratios and low observation counts.

The analyses of power resemble the results of the AUC comparison and are depicted in Additional file 1: Fig. S3. The type I error rates for the tree-based methods seem to randomly scatter around the prespecified significance level of 5%. However, the elastic net induces type I error rates of around two percent and is, therefore, quite conservative. The corresponding type I error rates are shown in Additional file 1: Fig. S4.

In Additional file 1: Figs. S5–S7, the results for the accuracy, sensitivity, and specificity are depicted. The accuracies resemble the results of the AUC evaluation, while the sensitivities and specificities do not show a clear pattern between the evaluated methods. These figures also show that, for increasing odds ratios, the specificities increase while the sensitivities decrease.

We also evaluated the GRS on the training data itself to compare the degrees of overfitting. Here, ordinary random forests leads to the severest overfitting. For data with high statistical noise and small effect sizes, its AUC almost reaches 100% compared to the true AUC of around 56%. The other tree-based algorithms also induce higher training AUCs than the true model, but not larger than random forests. In particular, a prior variable selection can indeed reduce the intensity of overfitting. The elastic net yields in most cases the lowest values for the AUC closely following the AUCs of the true model. Taking the test data analyses into account, this indicates a mixture of underfitting and slight overfitting of the elastic net. The training data results can be found in Additional file 1: Fig. S8.

#### Dominant interaction effects of SNPs

**Fig. 4:**
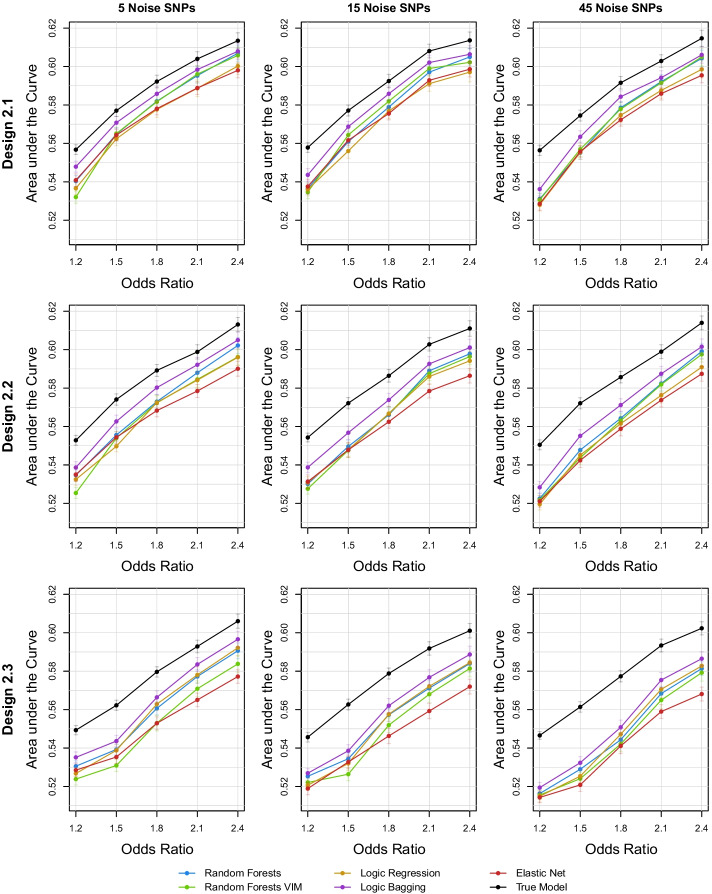
Mean AUC for random forests, random forests VIM, logic regression, logic bagging, elastic net, and the true underlying model in the second simulation scenario incorporating interactions of SNPs evaluated on the test data. The Designs 2.1, 2.2, and 2.3 describe the scenarios where both interacting SNPs also exhibit marginal effects, only one of both SNPs shows a marginal signal or none of them induce a main effect, i.e., (j, k) = (1, 2), (1, 4), or (4, 5) in Eq. (3), respectively

For the analysis of the scenarios with influential interaction terms, the performances of the statistical learning procedures measured by the AUC are shown in Fig. 4. Additionally, asymptotic 95% confidence intervals can be found in Additional file 1: Fig. S9. Similar to the main effects scenarios, logic bagging induces in each scenario the highest values of the AUC. Also as in the other settings, random forests VIM does not gravely suffer from noisy data compared to standard random forests, but cannot severely outperform its ordinary counterpart. Random forests itself seems to be the second-best performing method with an almost steady but close distance to logic bagging. Interactions of variables without marginal effects seem to be less of an issue to conventional logic regression, since for Design 2.3 and larger interaction effect sizes, logic regression achieves comparable AUCs to random forests. For weak interaction effects, the elastic net can yield comparative results to random forests and the logic regression. Nonetheless, increasing the interaction effect also increases the discrepancy between the tree-based approaches and the elastic net.

The results of the corresponding power and type I error analyses can be found in Additional file 1: Figs. S10 and S11. As in the previous simulation scenario, the comparison of the estimates of the statistical power resembles the corresponding analyses of the AUC. Again, the type I error rates for the tree-based methods seem to randomly scatter around 5%, whereas the elastic net leads to substantially lower error rates.

The results for the accuracy, sensitivity, and specificity can be found in Additional file 1: Figs. S12–S14. Similar to the marginal effects simulation scenario, the comparisons of the mean accuracy resemble the results of the AUC evaluation. The other two metrics sensitivity and specificity do not yield clear patterns between the considered procedures.

Evaluations of the GRS on the training data reveal again that conventional random forests seems to induce the severest overfitting. The results of these training data set applications are summarized in Additional file 1: Fig. S15.

#### Gene-environment interactions

**Fig. 5:**
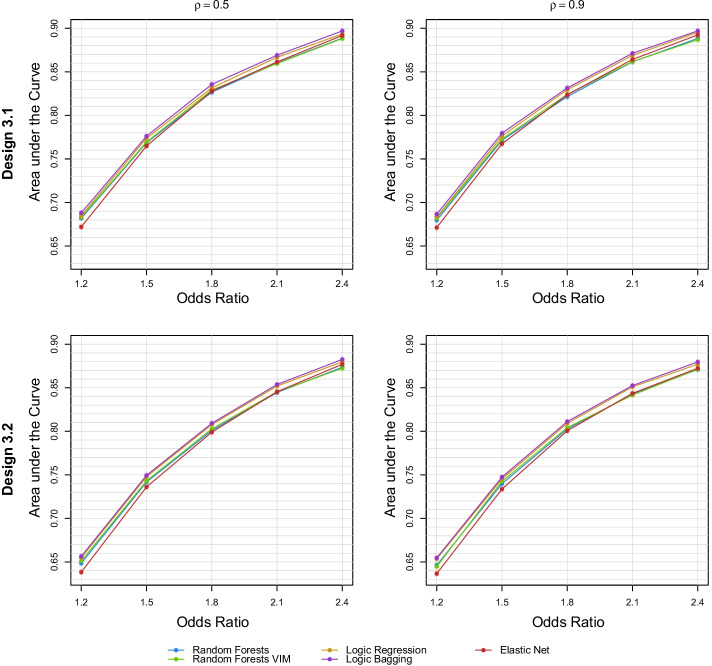
Mean AUC for random forests, random forests VIM, logic regression, logic bagging, and elastic net in the third simulation scenario incorporating continuous input variables evaluated on the test data. The Designs 3.1 and 3.2 describe the scenarios where the GxE interacting SNP also exhibits a moderate marginal effect or where it does not induce a main effect, i.e., j = 2 or 5 in Eq. (4), respectively

Figure 5 depicts the predictive performances of the statistical learning procedures for the 20 settings in the GxE interaction simulation scenario. Corresponding asymptotic 95% confidence intervals are shown in Additional file 1: Fig. S16. In contrast to the previous scenario, a true unique GRS model does not exist, since the GRS is based only on the genetic data while the true model of this scenario also consists of environmental covariables. Similar to the gene-gene interaction scenario, logic bagging leads in each setting to the highest AUCs. Throughout all settings in this simulation scenario, logic regression seems to be the second best performing method yielding AUCs closely below the AUCs of logic bagging. Random forests and random forests VIM induce very similar results such that there is no clear pattern between these two methods. For weak GxE interaction effects, the elastic net induces comparably poor results. However, for increasing GxE interaction effects, the discrepancy between random forests and elastic net decreases such that, for an odds ratio of 2.4, the elastic net yields slightly higher AUCs than random forests which are, however, still below the AUCs of logic bagging.

The correlation $\rho$ of the two continuous variables does not seem to affect the GRS performance in this simulation scenario. Nonetheless, the overall performance in Design 3.1 is higher than the performance in Design 3.2. This phenomenon can be explained by the absence of a marginal effect of the GxE interacting SNP in Design 3.2 complicating the identification of this SNP.

For this simulation scenario, the statistical power for all considered methods and simulation settings was equal to 100%. Similar to the previous scenarios, the elastic net seems to be more conservative as it induces lower type I error rates than the tree-based methods. The estimated type I error rates can be found in Additional file 1: Table S1.

In Additional file 1: Fig. S17–S19, the results for the accuracy, sensitivity, and specificity are depicted. Similar to the power analyses, the mean accuracies of the considered methods are almost identical in each simulation setting. However, for weak GxE interaction effects, the elastic net seems to induce the lowest mean accuracies. The results for the other two metrics, the sensitivity and the specificity, are also very similar.

Training data evaluations reveal again that conventional random forests tends to induce the severest overfitting. The training data results are depicted in Additional file 1: Fig. S20.

#### Comparison considering binary SNP codings

Additionally to considering the standard way of specifying the input variables for the different methods, we also evaluated the GRS construction approaches using the binary $\lbrace 0,1 \rbrace$ SNP coding for each method and not exclusively for logic regression and logic bagging. The detailed results for the $\lbrace 0,1 \rbrace$ SNP coding and the respective simulation scenarios are depicted in Additional file 1: Figs. S21–S23.

In comparison to using the $\lbrace 0,1,2 \rbrace$ coding, the performance of random forests and random forests VIM decreases. This is not very surprising, since, as pointed out in the methodological description, decision trees and random forests consider the dominant and recessive modes of inheritance when using the $\lbrace 0,1,2 \rbrace$ coding. Thus, using the $\lbrace 0,1 \rbrace$ coding doubles the number of input variables without supplying more information to random forests. The increase in the number of input variables complicates identifying the ideal splits when using typical settings for the hyperparameter $mtry$.

For the elastic net, the performance increases when employing the $\lbrace 0,1 \rbrace$ coding instead of the conventional $\lbrace 0,1,2 \rbrace$ coding such that, in the marginal effects simulation scenario and in the GxE interaction scenario, the elastic net yields similar results as logic bagging when considering settings with stronger genetic effects. Nonetheless, in the gene-gene interaction simulation scenario for the Designs 2.2 and 2.3 in which at least one interacting SNP does not exhibit a marginal effect, the elastic net with the $\lbrace 0,1 \rbrace$ SNP coding still induces inferior AUCs compared to logic bagging.

## Real data application

We also compared the GRS construction approaches using a real data set from a German cohort study, the SALIA study (**S**tudy on the Influence of **A**ir Pollution on **L**ung, **I**nflammation and **A**ging) [54], which included in total 4874 women that were at their first examination between 54 and 55 years old. The participants were recruited in 1985-1994 from highly industrialized areas and less industrialized areas in North-Rhine Westphalia, Germany. In 2006, a follow-up questionnaire was completed by 4027 women which contained questions about the diagnosis of certain diseases. In a further follow-up clinical examination conducted in 2007-2010, genetic data was also gathered. Here, we considered a data set consisting of 517 women from the SALIA study for which the presence of rheumatic diseases and genetic data are available. Furthermore, information about the exposure to specific air pollutants, i.e., nitrogen dioxide (${\mathrm {NO}}_2$), nitrogen oxide [nitrogen monoxide ${\mathrm {NO}}$ and nitrogen dioxide ${\mathrm {NO}}_2$] (${\mathrm {NO}}_x$), particulate matter with an aerodynamic diameter of $\le 2.5 \mu m$ or $\le 10 \mu m$ (${\mathrm {PM}}_{2.5}$ or ${\mathrm {PM}}_{10}$), particulate matter with diameters of $2.5-10 \mu m$ (${\mathrm {PM}}_\mathrm {coarse}$), and the reflectance of ${\mathrm {PM}}_{2.5}$ filters (${\mathrm {PM}}_{2.5\ {\mathrm {absorbance}}}$), is available at the time of performing the examinations in 2008. The assessment of the exposure to air pollution was conducted as part of the ESCAPE (**E**uropean **S**tudy of **C**ohorts for **A**ir **P**ollution **E**ffects) project using land-use regression models [55, 56]. We used these air pollution exposures to assess GxE interactions. Information on covariables such as the BMI (body mass index), age, education status, smoking status, or workplace exposure for adjusting the final models is also available. In the questionnaire, it was asked whether any rheumatic disease was diagnosed. Thus, we considered prevalent rheumatic diseases as outcome in our analyses. Details on the SALIA study and the assessment of air pollution in this study are given by Krämer et al. [57] and Hüls et al. [58].

### Selection of relevant genetic factors

In order to construct proper GRS for genes potentially having an impact on the development of rheumatic diseases, we selected several genes which showed to be influential in a literature research. For the selection of relevant genes, we mainly focused on rheumatoid arthritis, since it is the most common rheumatic disease besides osteoarthritis [59–61].

In around 70% to 90% of rheumatoid arthritis patients, anti-citrullinated peptide antibodies (ACPA) can be detected [62]. For ACPA-positive rheumatoid arthritis, many identified genetic associations belong to the human leukocyte antigen (HLA) class II complex [63]. Thus, we selected genes from the HLA class II complex for which associations with rheumatoid arthritis have been detected. In particular, we chose the HLA-DRB1 gene which presumably explains a large portion of the heritability of rheumatoid arthritis in the HLA class II complex [63–66]. Furthermore, we included the HLA-DPB1 and HLA-DOA genes which also might influence the risk of developing rheumatoid arthritis [66–68].

Since we started by including all available SNPs within the respective genes, 385 SNPs from the three genes formed our basis which we reduced by exploiting high states of LD. Using PLINK version 1.9 [69, 70], we performed LD-based clumping [71] (considering $r^2 = 0.5$). This procedure resulted in 72 tag SNPs which were used to construct the GRS.

We also constructed genome-wide GRS based on a recent meta-analysis of GWAS regarding rheumatoid arthritis [72]. In this meta-analysis, only non-HLA loci were considered in contrast to the gene-based selection. 70 of the proposed SNPs were available in our data and were used to fit the GRS models.

### Gene-environment interaction analysis

Additionally, we also analyzed GxE interaction effects. For the risk of developing ACPA-positive rheumatoid arthritis, GxE interactions between HLA class II alleles and smoking have been discovered [73, 74]. It might be of interest if traffic-related air pollution also interacts with genetic risk factors in the development of rheumatoid arthritis. Thus, our logistic regression models for the evaluation of GRS have the shape

6 $$\begin{aligned} {\mathrm {logit}}({\mathbb {P}}(Y = 1)) = \beta _0 + \beta _1 \cdot {\mathrm {GRS}} + \beta _2 \cdot E + \beta _3 \cdot {\mathrm {GRS}} \cdot E + \sum _{i=1}^l \gamma _i \cdot C_i \end{aligned}$$

for the environmental variable *E* and covariables $C_1, \ldots , C_l$.

The selection of potential relevant covariables was performed in two steps. First, we applied a stepwise logistic regression with the AIC (Akaike information criterion) as the selection measure. This lead to the inclusion of the age, the BMI, the current smoking status, and the former smoking status. Next, we regarded this selection of variables in the final models jointly with the GRS and air pollutants. We excluded covariables which worsened the models, i.e., which lead to lower AUCs. After this procedure, only the age was left.

### Analysis of association and predictive strength

The analysis was conducted in a repeated train-test split scheme. For 100 repetitions, we randomly divided the whole data set into 50% training data and 50% test data similar to Hüls et al. [11]. The respective training data sets were further randomly divided into 75% training data for hyperparameter tuning and 25% validation data (for the considered values of the hyperparameters, see “Section Hyperparameter optimization”). The best performing hyperparameter setting across the average of these 100 validation iterations was chosen.

### Results of the real data application

A descriptive summary of the most important variables gathered in the data set from the SALIA study is given by Table 5. Most noticeably, we considered an unbalanced data set with 394 controls and 123 cases considering prevalent rheumatic diseases.

**Table 5:** Descriptive statistics of the regarded data set from the SALIA study stratified according to the status of rheumatic diseases

Variable		Controls	Cases
*N*		394	123
Mean age	[years] ± sd	$70.87 \pm 3.16$	$71.50 \pm 2.96$
Mean BMI	[kg/m^2^] ± sd	$26.42 \pm 3.93$	$27.46 \pm 3.86$
*N* Currently smoking		$21\ (5.44\%)$	$5\ (4.07\%)$
*N* Formerly smoking		$61\ (15.80\%)$	$15\ (12.20\%)$
Mean pack-years of smoking	[years] ± sd	$3.78 \pm 10.92$	$2.85 \pm 9.25$
Mean ${\mathrm {NO}}_2$	[μg/m^3^] ± sd	$26.66 \pm 7.34$	$27.94 \pm 7.69$
Mean ${\mathrm {NO}}_x$	[μg/m^3^] ± sd	$41.34 \pm 17.71$	$44.10 \pm 17.68$
Mean ${\mathrm {PM}}_{10}$	[μg/m^3^] ± sd	$26.99 \pm 2.16$	$27.39 \pm 2.42$
Mean ${\mathrm {PM}}_{\mathrm {coarse}}$	[μg/m^3^] ± sd	$9.52 \pm 1.66$	$9.81 \pm 1.84$
Mean ${\mathrm {PM}}_{2.5}$	[μg/m^3^] ± sd	$17.94 \pm 1.38$	$18.23 \pm 1.50$
Mean ${\mathrm {PM}}_{2.5 \ {\mathrm {absorbance}}}$	[μg/m^3^] ± sd	$1.47 \pm 0.46$	$1.58 \pm 0.59$

#### Univariate regression models

In the analysis of the data of the SALIA study, Table 6 summarizes the median p-values of GRS analyzed in univariate regression models as in Eq. (5). When testing the influence of the GRS on the risk of developing rheumatoid arthritis, conventional random forests and logic bagging are the only models achieving significance at a significance level of 5% for at least 50% of the evaluations.

**Table 6:** Median p-values of the Wald tests for univariate models only including the GRS built on the SALIA data set

Algorithm	Median *p* value
Random forests	0.018
Random forests VIM	0.167
Logic regression	0.353
Logic bagging	0.021
Elastic net	0.512

**Fig. 6:**
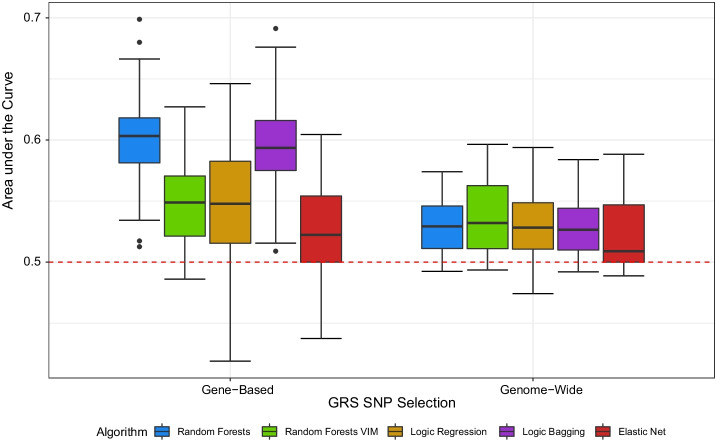
AUC for random forests, random forests VIM, logic regression, logic bagging, and elastic net in the application to data from the SALIA study evaluated on the test data. Results for single unadjusted models also considering the alternative genome-wide construction approach

Figure 6 summarizes the test AUC values for the tree-based statistical learning procedures and elastic net induced by univariate regression models only based on the GRS. For the gene-based approach, most noticeably, random forests and logic bagging yield the highest AUCs where random forests achieves a slightly better performance than logic bagging. Ordinary logic regression and random forests with a prior variable selection induce similar results which cannot compete with conventional random forests and logic bagging. However, the elastic net yields the lowest AUCs. Here, the lower quartile of the AUCs yielded by the elastic net reaches 50%, i.e., the predictive performance of a (non-informative) constant classifier.

In addition to gene-based GRS, we also constructed genome-wide GRS based on a recent GWAS meta-analysis regarding rheumatoid arthritis [72]. A specific comparison of the predictive power between the gene-based and genome-wide approaches is summarized in Fig. 6. However, for the genome-wide selection of SNPs, barely a signal can be observed in our sample as the AUCs on the test data sets were close to 50%. Thus, the genome-wide GRS construction approach was not included in subsequent analyses. The inferior predictive performance compared to the gene-based selection is possibly caused by the exclusion of HLA genes in the underlying meta-analysis. Nonetheless, the elastic net induces the lowest values for the AUC compared to the tree-based methods which is in line with our previous experiments. In contrast to the gene-based approach, random forests VIM yields a predictive power that can compete with ordinary random forests and logic bagging.

#### Gene-environment interaction analysis

In the final adjusted models of the form as in Eq. (6), we regarded each air pollutant indicator separately and included the respective GxE interaction term. Neither the GRS themselves nor the GxE interaction terms are significant at a significance level of 5%. The concrete median p-values of the 100 repetitions for the final adjusted models can be found in Additional file 1: Table S2.

**Fig. 7:**
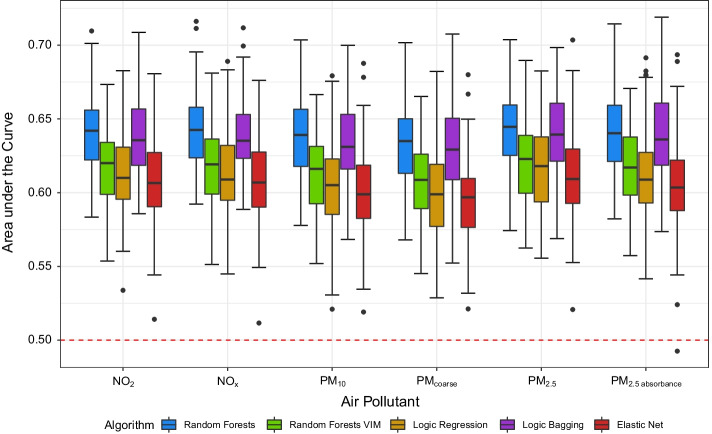
AUC for random forests, random forests VIM, logic regression, logic bagging, and elastic net in the application to data from the SALIA study evaluated on the test data. Results for the final age-adjusted models with different air pollution indicators

Figure 7 depicts the predictive performance of the considered statistical learning algorithms for the induction of gene-based GRS in multivariate regression models. Analogously to the univariate analysis, random forests and logic bagging yield the highest predictive power where the overall best values are reached for ${\mathrm {PM}}_{2.5}$. For this air pollutant, random forests achieves the best performance. The elastic net, random forests VIM, and logic regression yield similar performances which, again, cannot compete with random forests and logic bagging.

We also evaluated the GRS on the training data sets themselves. The best performing procedures random forests and logic bagging tend to heavily overfit the data as can be seen by the high discrepancy between the test and the training data analyses. These two algorithms achieve training AUCs of nearly 100% whereas the other methods lead to more homogeneous results. The corresponding AUCs can be found in Additional file 1: Fig. S24.

Smoking is a major risk factor for rheumatoid arthritis [75]. As can be seen in Table 5, the fractions of current smokers and former smokers in the excerpt from the SALIA study are higher among controls than among cases which is in contradiction to the literature. Since only 19.7% of the study participants in the data excerpt are current or former smokers, we conducted a sensitivity analysis excluding all current and former smokers from the data. Again, we are not able to identify any significant GxE interactions. The resulting AUCs are very similar to the former analysis. Random forests and logic bagging yield the highest test AUC values, whereas elastic net induces substantially lower values. The concrete results can be found in Additional file 1: Fig. S25.

## Discussion

In this analysis, we evaluated tree-based statistical learning approaches for the construction of GRS. We used the elastic net as a reference model and analyzed the tree-based statistical learning methods in a simulation study considering several scenarios, focusing on marginal and epistatic genetic effects, respectively. To confirm our findings, we constructed and assessed GRS on a real data set from the German SALIA cohort study.

As our analyses showed, a modification of logic regression, namely logic bagging, was able to outperform the reference GRS construction procedure, the elastic net, in almost every scenario of the simulation study.

Similarly, logic bagging lead to a comparably strong predictive performance in the real data application. Logic regression could only compete when considering large effect sizes in the simulation studies and yielded inferior results in the analysis of the SALIA data. This indicates that logic regression fits highly variant models which can indeed benefit from a variance reduction via an ensemble approach like bagging. For larger genetic effects, bagging does not seem to be necessary due to a more consequent identification of the underlying signal.

Random forests lead to the best predictive performance on the real data set. Considering the simulation study, in a likewise comparable scenario, i.e., small data sets, low marginal genetic effects, and higher amounts of statistical noise, random forests could induce comparably high values for the AUC as well. In the analysis of marginal genetic effects, random forests’ performance decreased for increasing amounts of noise. This phenomenon can be partly explained by the random selection scheme of predictors for partitioning. The input variables are drawn with equal probabilities without replacement. Therefore, considering the setting with 44 noise SNPs in the first simulation scenario, in a decision tree branch where already three of the six influential SNPs and no noise are included, the probability of regarding one of the three remaining influential SNPs for the next split with the standard setting $mtry = \lfloor \sqrt{50} \rfloor = 7$ is about only 39%. Thus, choosing a set of SNPs containing only statistical noise is more likely in this case. We also allowed higher settings for $mtry$ in the hyperparameter optimization as could be seen in Table 4. For higher amounts of statistical noise, the higher setting for $mtry$ could in fact increase the performance of random forests.

A related issue was the high amount of overfitting by random forests which could be observed in all three simulation scenarios as well as in the real data application. We addressed this by considering minimum terminal node sizes of up to 10% of the number of observations in each leaf and by performing a prior variable selection based on variable importance measures. The former solution, i.e., the tuning of the minimum node size, was important to optimize the performance on the general population, since the standard setting is set to one observation for classification trees. However, for appropriate probability estimates, Malley et al. [35] recommend choosing 10% of the total sample size.

The latter approach, i.e., the usage of random forests VIM, needed higher amounts of statistical noise and stronger marginal genetic effects to achieve test data performances comparable to random forests. Nonetheless, this alternative approach could substantially reduce the amount of overfitting in any case. Presumably caused by weak individual genetic effects, random forests VIM yielded an inferior predictive performance compared to ordinary random forests on the application to the SALIA data. However, in the analyses conducted by Speiser et al. [76], the random forests VIM approach utilizing the Boruta variable selection was able to yield lower error rates than conventional random forests. Thus, studies specifically comparing random forests variable selection procedures with conventional random forests in low signal-to-noise ratio scenarios, such as applications considering SNP data, might be beneficial.

The reference procedure, the elastic net, could not compete with logic bagging and random forests when considering stronger gene-gene interaction effects. Even for solely marginal genetic effects, the regularization procedure had difficulties achieving AUCs as high as the ones of logic bagging. However, for strong GxE interaction effects, the elastic net could induce similar predictive performances as random forests. Before deciding to choose the penalty parameter $\lambda$ based on the minimum cross-validation error, we evaluated the elastic net based on the maximum $\lambda$ which yielded a cross-validation error in the range of one standard error of the minimum error. This approach is also recommended by Waldmann et al. [77] for GWAS-level amounts of SNPs and used by Hüls et al. [49] for the construction of GRS. However, in our applications including both the simulation study and the real data application, the elastic net had difficulties recognizing a signal at all with this approach which was presumably caused by high errors in general. Thus, we chose the minimizing $\lambda$ which enhanced our fitted elastic net models.

In practice, the conventional $\lbrace 0,1,2 \rbrace$ SNP coding is utilized when constructing GRS with regularized regression approaches such as the elastic net [11, 16]. Thus, we focused on this standard procedure in our analyses, which lead to comparatively weak performances. However, when splitting each considered SNP into two binary variables, i.e., when using the binary $\lbrace 0,1 \rbrace$ SNP coding also for the elastic net, its performance in the simulation study increased due to now being able to differentiate between the dominant and recessive modes of inheritance. Therefore, the results for the $\lbrace 0,1 \rbrace$ SNP coding suggest that it might be preferable to employ the $\lbrace 0,1 \rbrace$ coding when fitting GRS using the elastic net. Nonetheless, logic bagging still yielded higher predictive performances than the elastic net in the gene-gene interaction simulation scenario when considering the $\lbrace 0,1 \rbrace$ coding for all procedures.

The most important advantage of the tree-based methods regarded in this article is to not being restricted to model assumptions such as linearity, i.e., being able to autonomously detect gene-gene interactions. The assumption of oversimplified genetic architectures in linear models might be the main cause for random forests and logic bagging outperforming the elastic net in most analyses. However, it is well known that gene-gene interactions also play a role in the heritability of diseases [8, 9].

Another practically interesting question would be, how well the introduced tree-based methods can construct GRS for significantly larger amounts of SNPs, e.g., when using a broader SNP selection from GWAS. Winham et al. [22] found in their studies that for increasing amounts of SNPs, the identification of interactions becomes more difficult for random forests. For logic regression, with increasing amounts of explanatory variables, the amount of possible states increases linearly, therefore, requiring more simulated annealing iterations and generally deeper greedy searches and, hence, increasing the model fitting time. This model building time must be further increased when considering higher values for the parameters of maximum trees and maximum leaves which is reasonable due to potentially more influential predictors for more total input variables.

Unsurprisingly, elastic net models could be fitted and evaluated in the least amount of time due to their simplicity compared to the considered tree-based models. Random forests with 2000 trees could be fitted and evaluated in less than 10 s in most cases. Random forests VIM needed slightly more time which was to be expected. Logic bagging models needed more time, however, conventional logic regression models utilizing simulated annealing as search procedure consumed the most amount of time and needed up to 1 minute for fitting and evaluating the GRS. In Additional file 1: Fig. S1, the concrete times for the third simulation scenario are depicted.

For increasing odds ratios, the measured sensitivity decreases in the marginal effects and gene-gene interaction effect simulation scenarios, which does not seem to be plausible at first glance. However, this phenomenon can be explained by the data structure considered in this analysis and the requirement to dichotomize the risk predictions into two classes for estimating the sensitivity and specificity. For constructing GRS, discrete input variables, more exactly SNPs exhibiting three different outcomes, are used. Thus, the constructed and possibly true underlying GRS also follow a discrete pattern depending on the SNP setting. For the marginal effects simulation scenario, there are 7 distinct GRS values in the true underlying model due to Eq. (2). In Additional file 1: Fig. S26, a corresponding GRS distribution is depicted. Due to the additivity in this model, the GRS just below 0.5 occurs in approximately 30% of all observations. Therefore, dichotomizing the GRS at 0.5 leads to classifying only 35% of all observations as cases which explains the low sensitivity in this setting. Lowering the classification threshold to a value such as 0.45 shifts the issue to the specificity, since, in this case, only 35% of all observations will be classified as controls. Thus, the sensitivities and specificities determined in this analysis need to be interpreted with caution because of the discrete nature of the considered input variables.

In our real data application, we analyzed a relatively small data set containing 517 observations with only 123 cases. The missing balance as well as the comparably low sample size complicated meaningful analyses, especially when considering the need for splitting the data set into training and test data sets. Generally, important covariates such as the smoking status and the BMI were not included in the final models due to lowering the predictive performance. This decrease in performance was presumably caused by the low sample size and amount of cases yielding unintuitive statistics such as the higher fraction of smokers among controls.

## Conclusion

As our analyses on simulated as well as on real data showed, the tree-based statistical learning methods random forests and logic bagging can be valuable tools for constructing GRS. Especially when little prior knowledge about the gene-response relationships is available or if no appropriate external weights for the regarded disease or population are available, these two algorithms should also be taken into consideration when building GRS. Regardless of the presence of gene-gene interactions in the heritability of a certain disease, the discussed methods have the potential to outperform regularized linear methods.

## Supplementary Information

**Additional file 1**. Further evaluation results, hyperparameter descriptions, and method workflows. Additional methodological descriptions and results for the simulation study and the real data application.

**Additional file 1**. Simulation study data generating code. R code for generating and accessing all data sets used in the simulation study.

## Data Availability

All code for generating and accessing data for the simulation study is included in this published article as a supplementary information file (Additional file 2).

## Abbreviations

ACPA: Anti-citrullinated peptide antibody
AIC: Akaike information criterion
AUC: Area under the curve
BMI: Body mass index
CART: Classification and regression tree
GLM: Generalized linear model
GRS: Genetic risk score(s)
GWAIS: Genome-wide association interaction study
GWAS: Genome-wide association study
GxE: Gene-environment
HLA: Human leukocyte antigen
IQR: Interquartile range
LD: Linkage disequilibrium
MAF: Minor allele frequency
ROC: Receiver operating characteristic
SNP: Single nucleotide polymorphism
VIM: Variable importance measure
