# Supplementary material for: Evaluation of tree-based statistical learning methods for constructing genetic risk scores
Source: BMC Bioinformatics. 2022 Mar 21;23:97. doi: 10.1186/s12859-022-04634-w (PMC8935722; doi:10.1186/s12859-022-04634-w)
Supplement: Supplementary file 1 — Additional file 1. Further evaluation results, hyperparameter descriptions, and method workflows. Additional methodological descriptions and results for the simulation study and the real data application. [file 12859_2022_4634_MOESM1_ESM.pdf]

Additional file 1

Evaluation of tree-based statistical learning methods for constructing genetic risk scores

Michael Lau[1,2,*], Claudia Wigmann[2], Sara Kress[2], Tamara Schikowski[2] and Holger Schwender[1]

[1] *Mathematical Institute, Heinrich Heine University, Düsseldorf, Germany*
[2] *IUF – Leibniz Research Institute for Environmental Medicine, Düsseldorf, Germany*
[*] *Correspondence: michael.lau@hhu.de*

In this supplementary file, additional information about the GRS construction methods and additional results about the simulation study and the real data application are presented. In Figure S1, model fitting and GRS prediction times are depicted. In Section 2, the considered hyperparameters for constructing the GRS models are described. In Section 3, we present the workflows for tuning and fitting each regarded statistical learning procedure for constructing GRS. Means and asymptotic 95% confidence intervals of the AUCs corresponding to the figures in the main text are depicted in the Figures S2, S9, and S16. Concrete estimates following statistical inference can be found in the Figures S3, S4, S10, S11, and in Table S1. Results for the classical classification metrics accuracy, sensitivity, and specificity are depicted in the Figures S5, S6, S7, S12, S13, S14, S17, S18, and S19. Training data AUCs are illustrated in the Figures S8, S15, S20, and S24. AUC comparisons when employing the binary $\{0, 1\}$ SNP coding for each method are depicted in the Figures S21, S22, and S23. Table S2 depicts median p-values of the final adjusted models for the GRS, the environmental factor, and their interaction term. Final results for the sensitivity analysis excluding smokers from the SALIA data set can be found in Figure S25. In Figure S26, an exemplary GRS distribution is depicted which explains the observed sensitivities in the simulation study.

1 Model fitting and GRS prediction time

We, here, present the model fitting and GRS prediction times in the third simulation scenario. The times for single model constructions and evaluations in the hyperparameter optimization process are presented, since, in the hyperparameter optimization process, several different settings, which can have an impact on the time, are utilized.

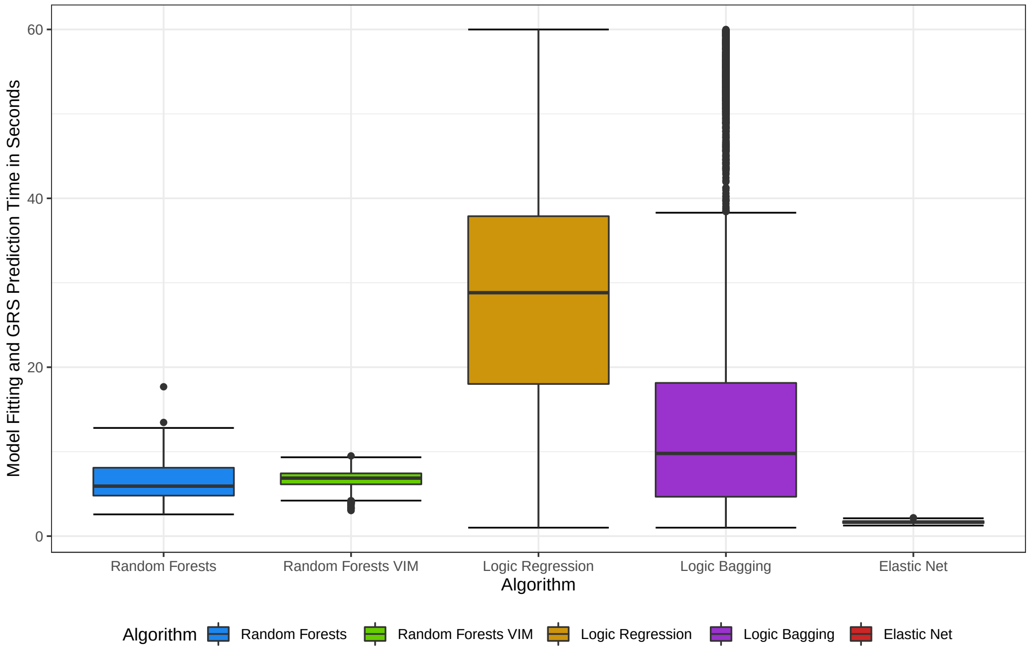

Figure S1: Model fitting and GRS prediction time for random forests, random forests VIM, logic regression, logic bagging, and elastic net for the hyperparameter configuration in the third simulation scenario incorporating continuous input variables.

2 Hyperparameter descriptions

We, here, briefly describe the hyperparameters of each considered statistical learning procedure that were tuned in our analyses. Table 4 in the main text depicts the corresponding hyperparameter settings.

2.1 Random forests & random forests VIM

The parameter mtry determines the number of randomly chosen input variables regarded at each split in each tree. The parameter min.node.size configures the number of observations which have to belong to a certain tree node in order to continue splitting this node. Thus, min.node.size acts as a stopping criterion for prematurely terminating splitting of a tree branch. num.trees determines the total number of trees to be grown in random forests. A sufficiently high number should be chosen such that the performance will not increase substantially anymore.

2.2 Logic regression & logic bagging

For logic regression and logic bagging, ntrees and nleaves determine the model complexity. ntrees is the maximum number of trees to be included in the model and nleaves is the maximum number of leaves distributed over all trees.

For conventional logic regression, simulated annealing is employed as the search algorithm which has to be tuned as well. For the number of simulated annealing iterations, analogously to the number of trees in random forests, a sufficiently high number should be chosen. The cooling schedule, which includes a start temperature and an end temperature, is manually tuned such that at the beginning of the search, almost all states are accepted, and at the end of the search, almost no states are accepted.

For logic bagging, the number of bagging iterations has to be set to a sufficiently high number, similar to num.trees and the number of simulated annealing iterations.

2.3 Elastic net

For fitting elastic net models, the parameter α controls the balance between the lasso and the ridge regularization. The parameter λ determines the strength of the regularization.

3 Tuning and training workflows

Since each statistical learning method regarded in this article requires considering different details for properly fitting GRS models, we here briefly present the workflows for each method.

3.1 Random forests

1. Choose a sufficiently high number of trees to be fitted, e.g., 2000
2. Tune the minimum node size and the number of randomly chosen predictors at each split in each tree using a grid search by fitting a random forest with probability estimation trees for each eligible setting
3. Fit a random forest with probability estimation trees using the best identified hyperparameter configuration

3.2 Random forests VIM

1. Choose a sufficiently high number of trees to be fitted, e.g., 2000
2. Tune the minimum node size and the number of randomly chosen predictors at each split in each tree using a grid search by performing a variable selection via the Boruta approach and fitting a random forest with probability estimation trees for each eligible setting
3. Perform a variable selection via the Boruta approach and fit a random forest with probability estimation trees using the best identified hyperparameter configuration

3.3 Logic regression

1. Split all considered SNPs into two binary variables coding for dominant and recessive effects
2. Choose a sufficiently high number of markov chain iterations to be executed, e.g., 500000
3. Experimentally tune the cooling schedule for simulated annealing, i.e., choose a start temperature such that almost all states are accepted and choose a final temperature such that almost no states are accepted

4. Tune the number of trees and the total number of leaves using a grid search by fitting a logic regression model with the logit link function for each eligible setting

5. Fit a logic regression model with the logit link function using the best identified hyperparameter configuration

3.4 Logic bagging

1. Split all considered SNPs into two binary variables coding for dominant and recessive effects

2. Choose a sufficiently high number of bagging iterations to be performed, e.g., 500

3. Tune the number of trees and the total number of leaves using a grid search by fitting a logic bagging model with the logit link function for each eligible setting. A logic bagging model is fitted by drawing a bootstrap sample and fitting a logic regression model with a greedy search to this sample for each bagging iteration.

4. Fit a logic bagging model with the logit link function using the best identified hyperparameter configuration

3.5 Elastic net

1. Tune the elastic net parameter α using a grid search by fitting an elastic net model with the logit link function for each eligible setting. Automatically configure the regularization parameter λ by performing an inner cross-validation (`cv.glmnet` in `glmnet`).

2. Fit an elastic net model with the logit link function using the best identified hyperparameter configuration

4 Simulation studies

4.1 Marginal genetic effects

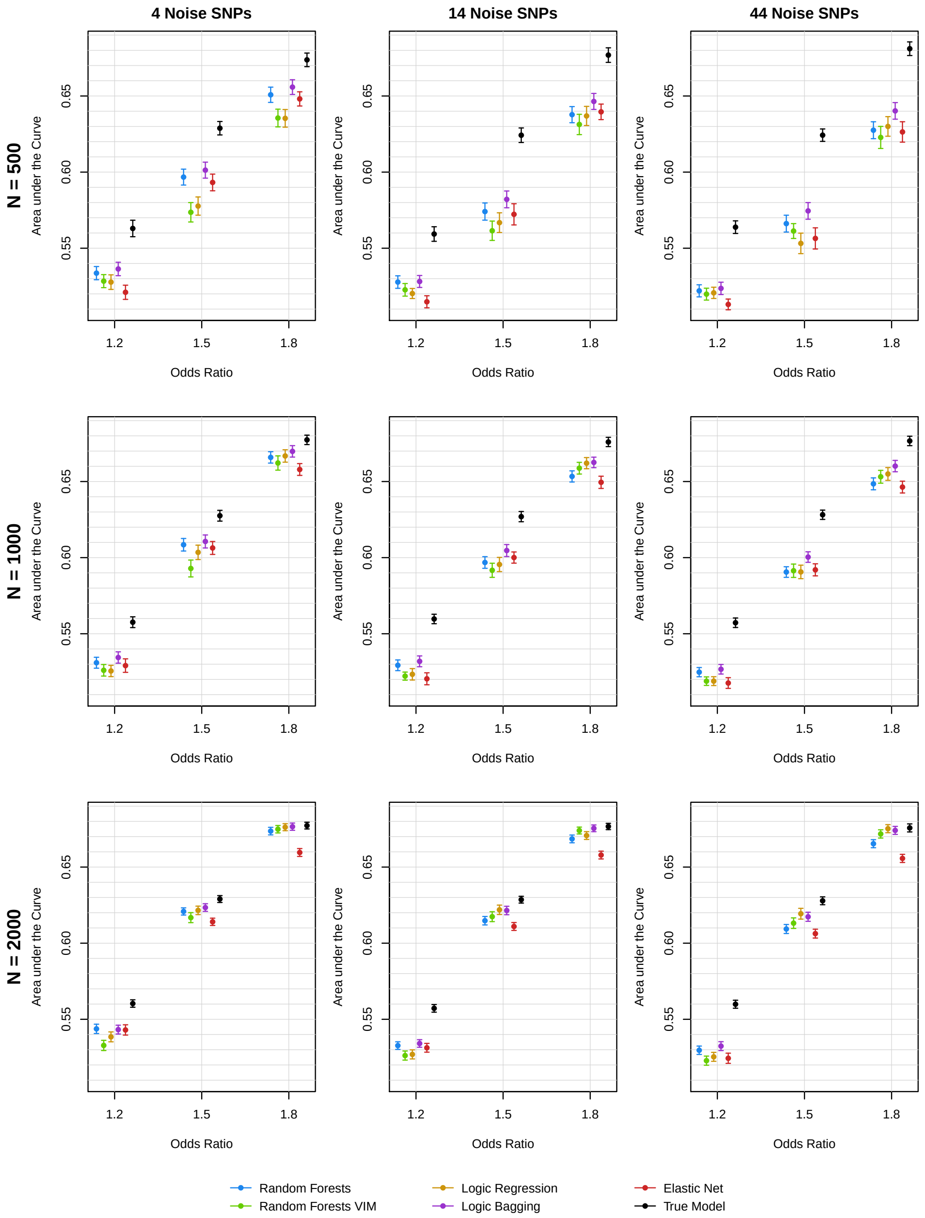

Figure S2: Mean AUC and asymptotic 95% confidence intervals for random forests, random forests VIM, logic regression, logic bagging, elastic net, and the true underlying model in the first simulation scenario considering marginal effective SNPs evaluated on the test data.

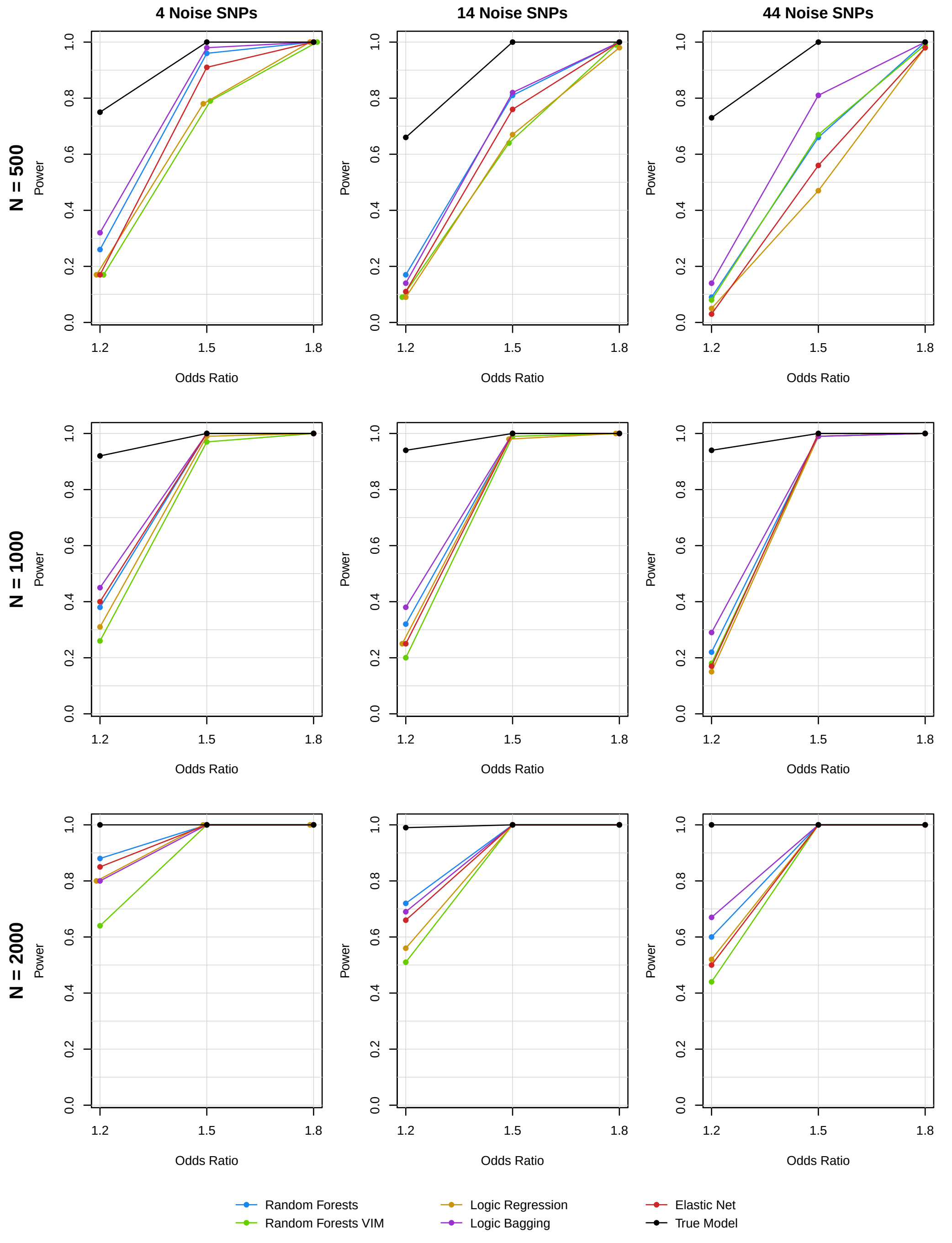

Figure S3: Estimated power for random forests, random forests VIM, logic regression, logic bagging, elastic net, and the true underlying model in the first simulation scenario considering marginal effective SNPs evaluated on the test data.

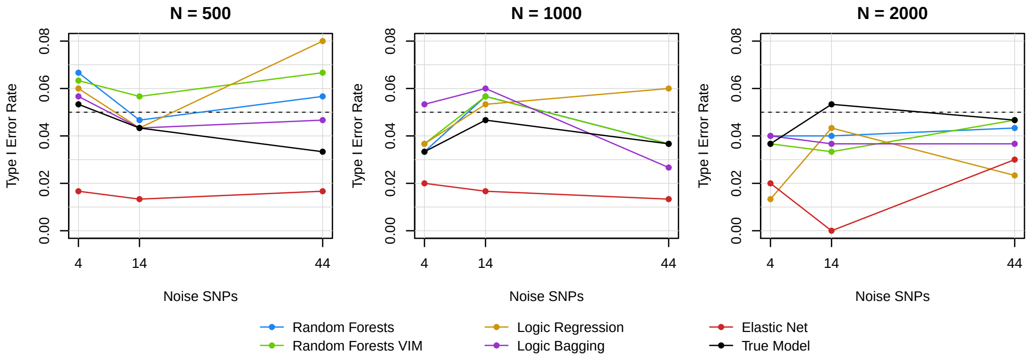

Figure S4: Estimated type I error rate for random forests, random forests VIM, logic regression, logic bagging, elastic net, and the true underlying model in the first simulation scenario considering marginal effective SNPs evaluated on the test data.

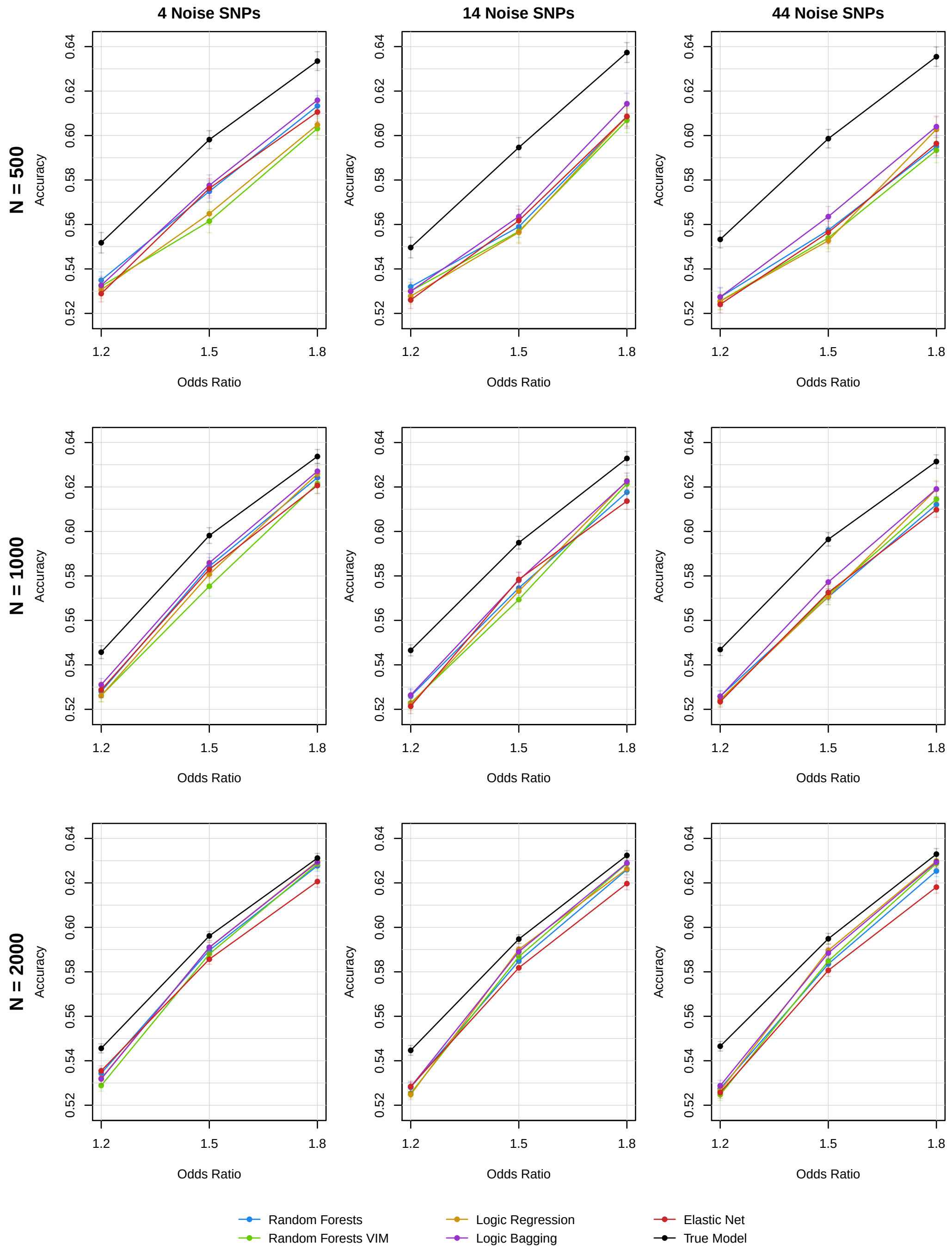

Figure S5: Mean accuracy for random forests, random forests VIM, logic regression, logic bagging, elastic net, and the true underlying model in the first simulation scenario considering marginal effective SNPs evaluated on the test data.

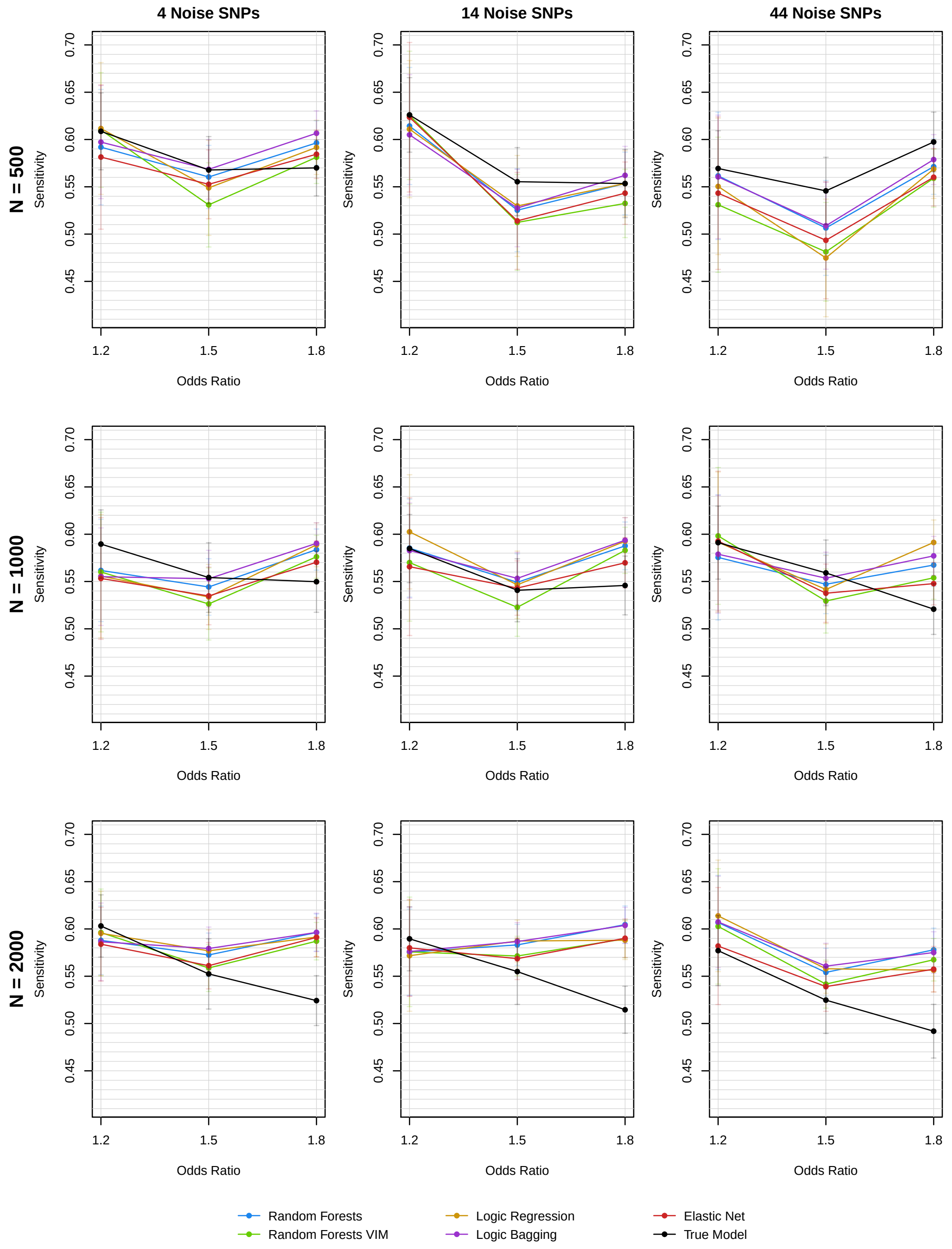

Figure S6: Mean sensitivity for random forests, random forests VIM, logic regression, logic bagging, elastic net, and the true underlying model in the first simulation scenario considering marginal effective SNPs evaluated on the test data.

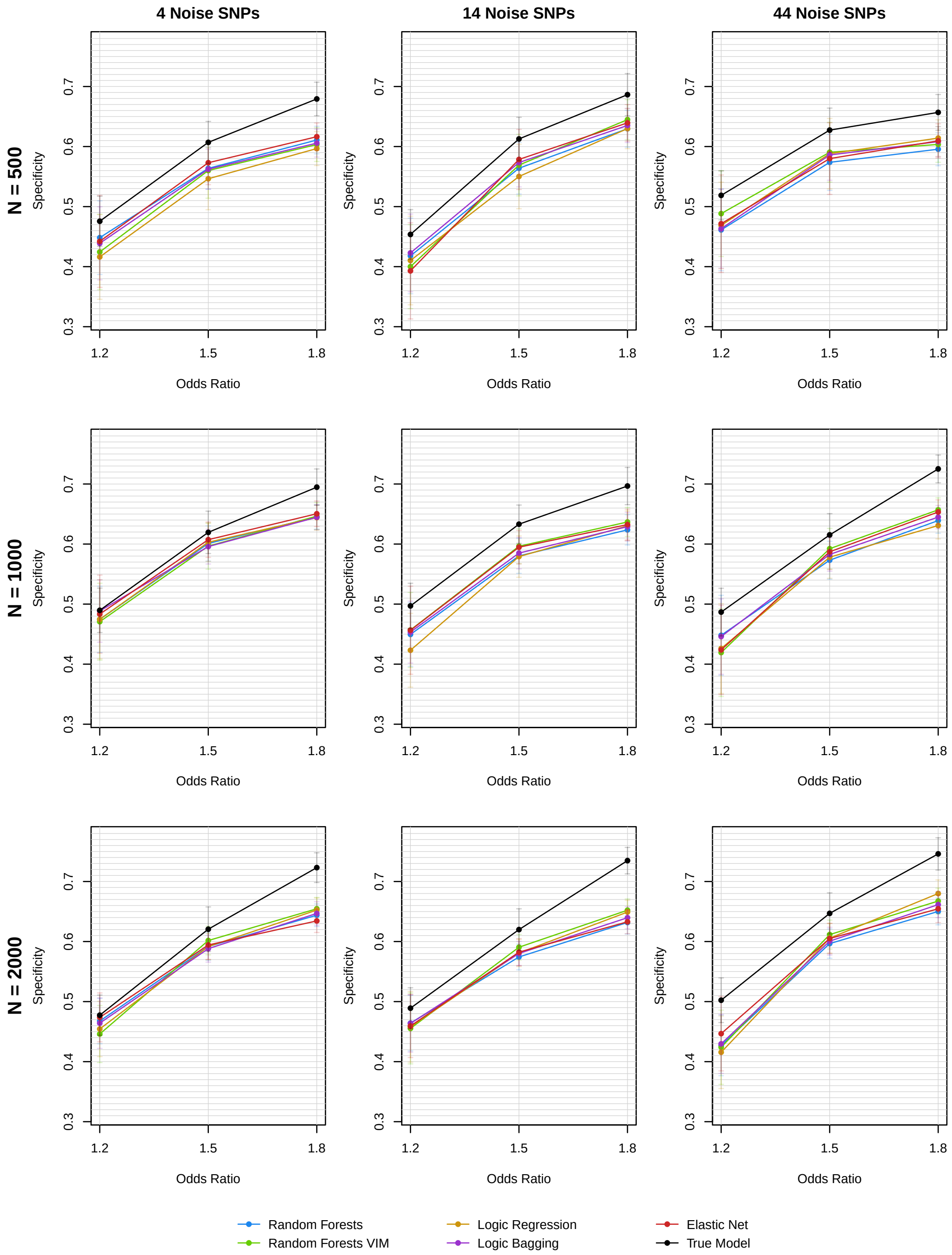

Figure S7: Mean specificity for random forests, random forests VIM, logic regression, logic bagging, elastic net, and the true underlying model in the first simulation scenario considering marginal effective SNPs evaluated on the test data.

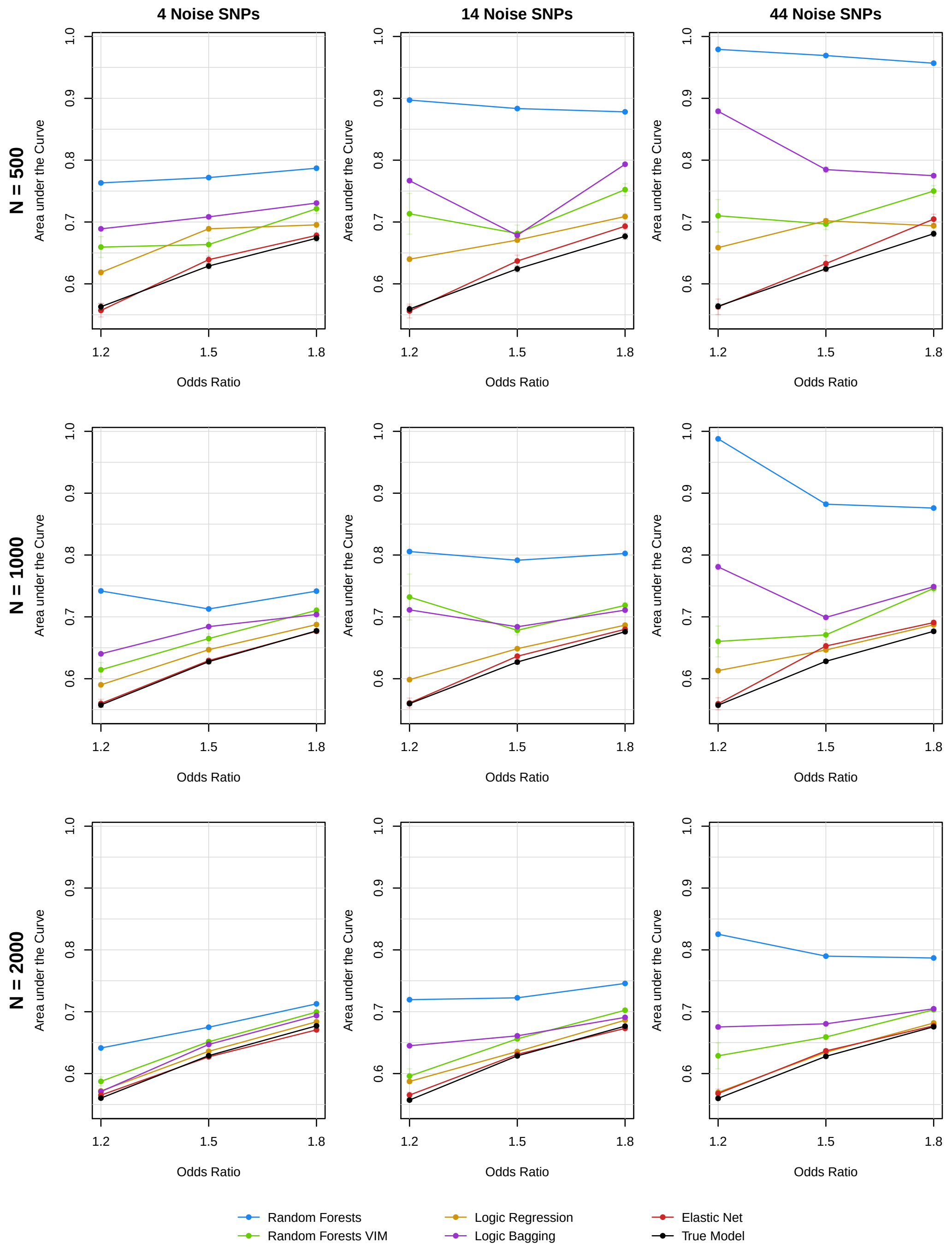

Figure S8: Mean AUC for random forests, random forests VIM, logic regression, logic bagging, elastic net, and the true underlying model in the first simulation scenario considering marginal effective SNPs evaluated on the training data itself.

4.2 Dominant interaction effects of SNPs

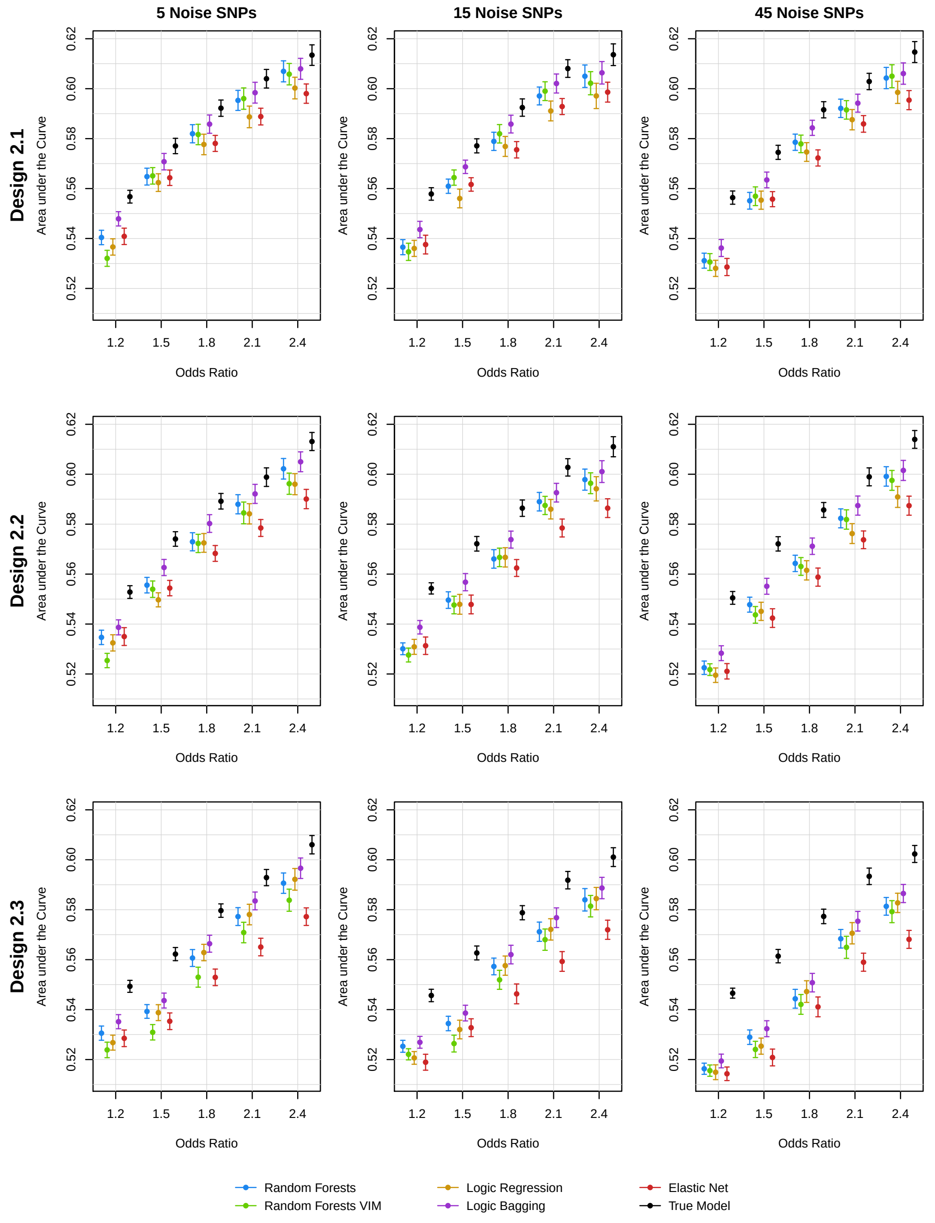

Figure S9: Mean AUC and asymptotic 95% confidence intervals for random forests, random forests VIM, logic regression, logic bagging, elastic net, and the true underlying model in the second simulation scenario incorporating interactions of SNPs evaluated on the test data.

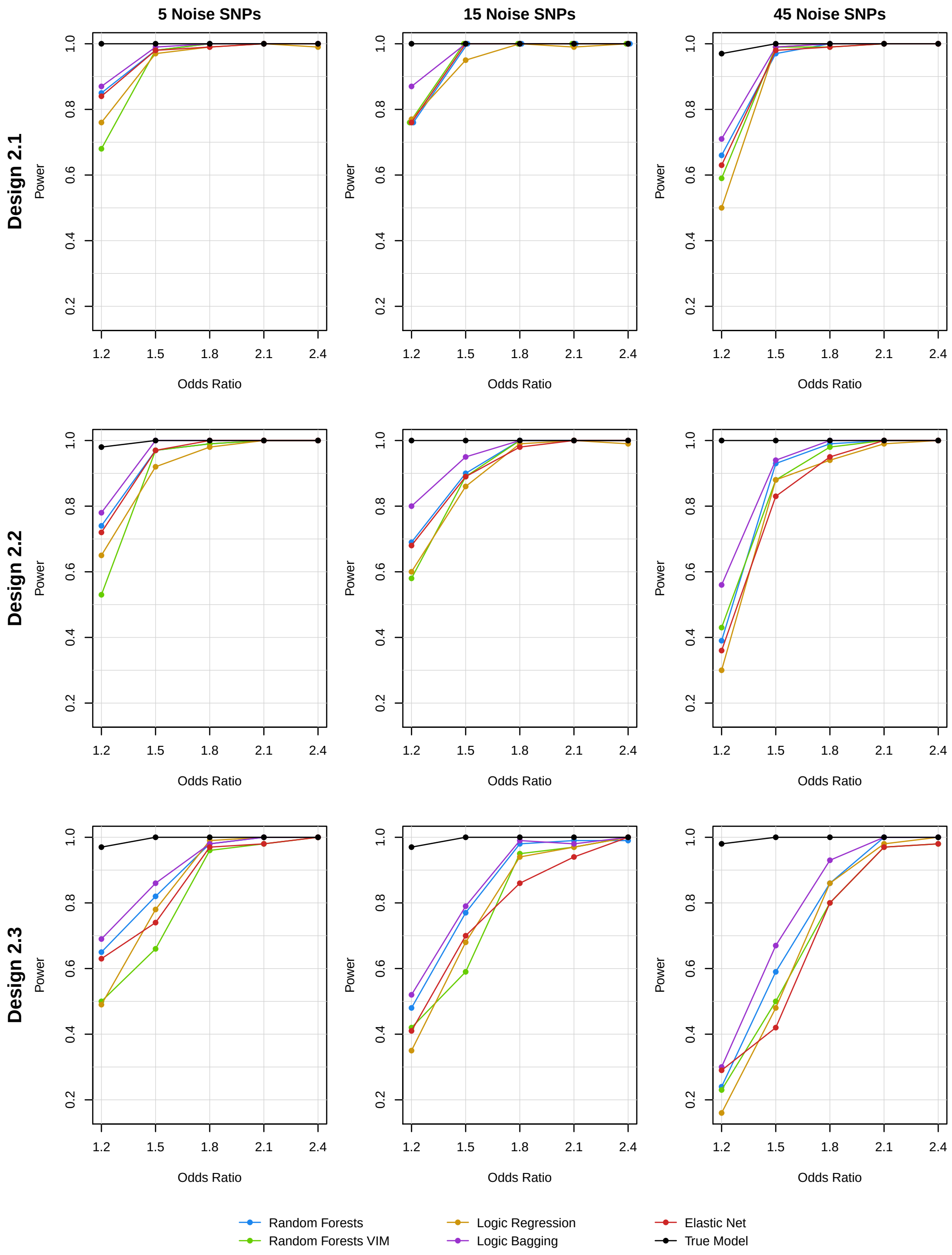

Figure S10: Estimated power for random forests, random forests VIM, logic regression, logic bagging, elastic net, and the true underlying model in the second simulation scenario incorporating interactions of SNPs evaluated on the test data.

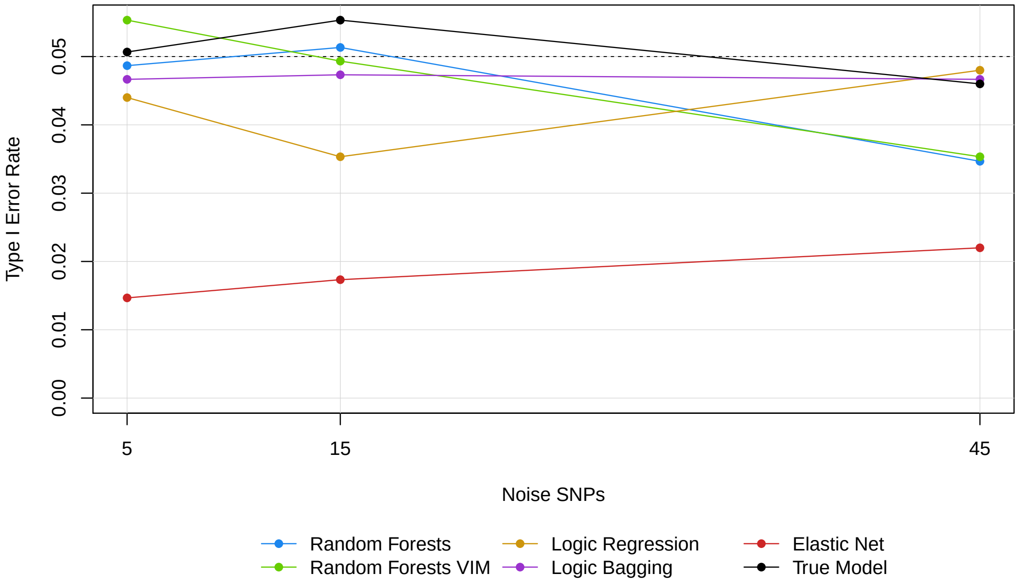

Figure S11: Estimated type I error rate for random forests, random forests VIM, logic regression, logic bagging, elastic net, and the true underlying model in the second simulation scenario incorporating interactions of SNPs evaluated on the test data.

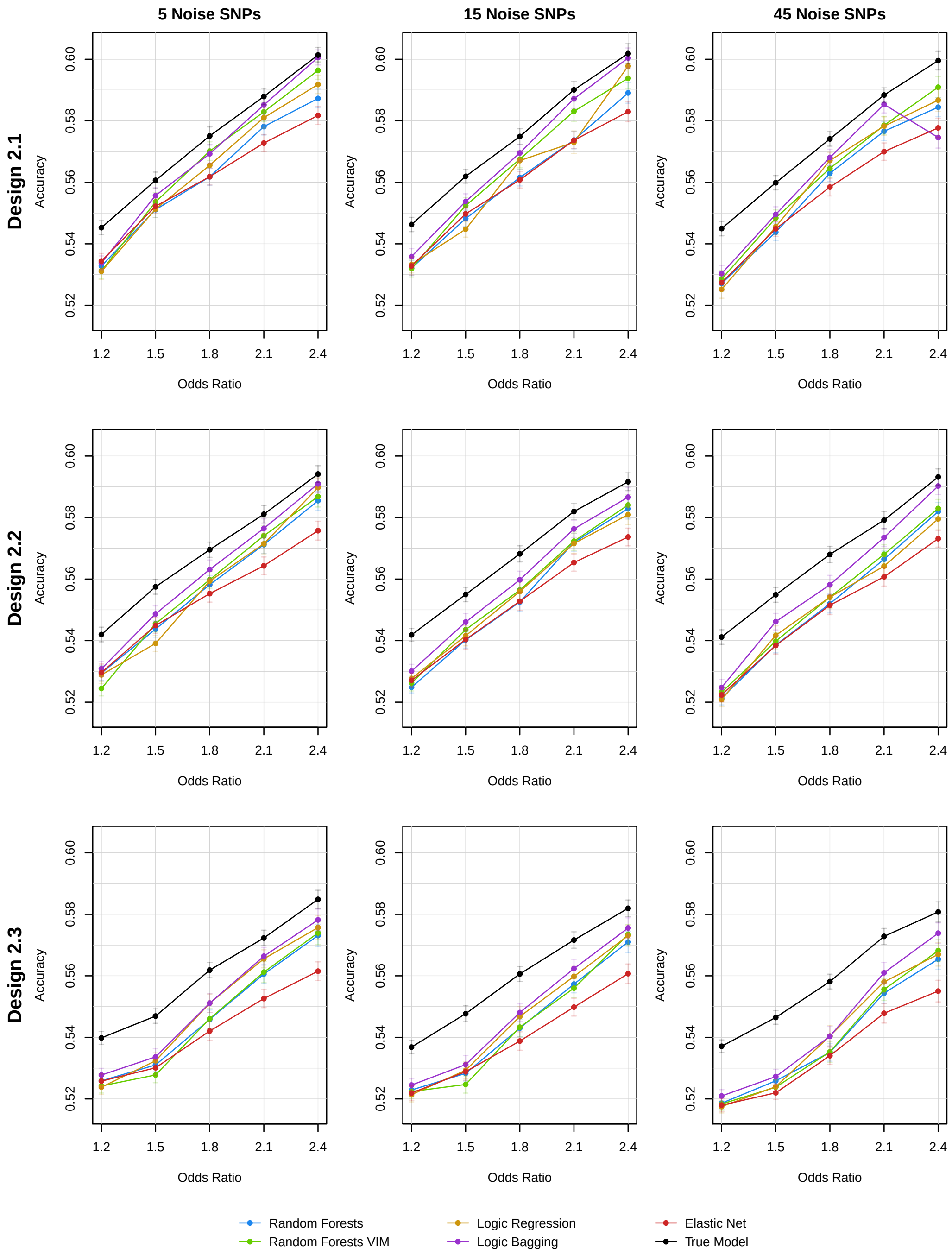

Figure S12: Mean accuracy for random forests, random forests VIM, logic regression, logic bagging, elastic net, and the true underlying model in the second simulation scenario incorporating interactions of SNPs evaluated on the test data.

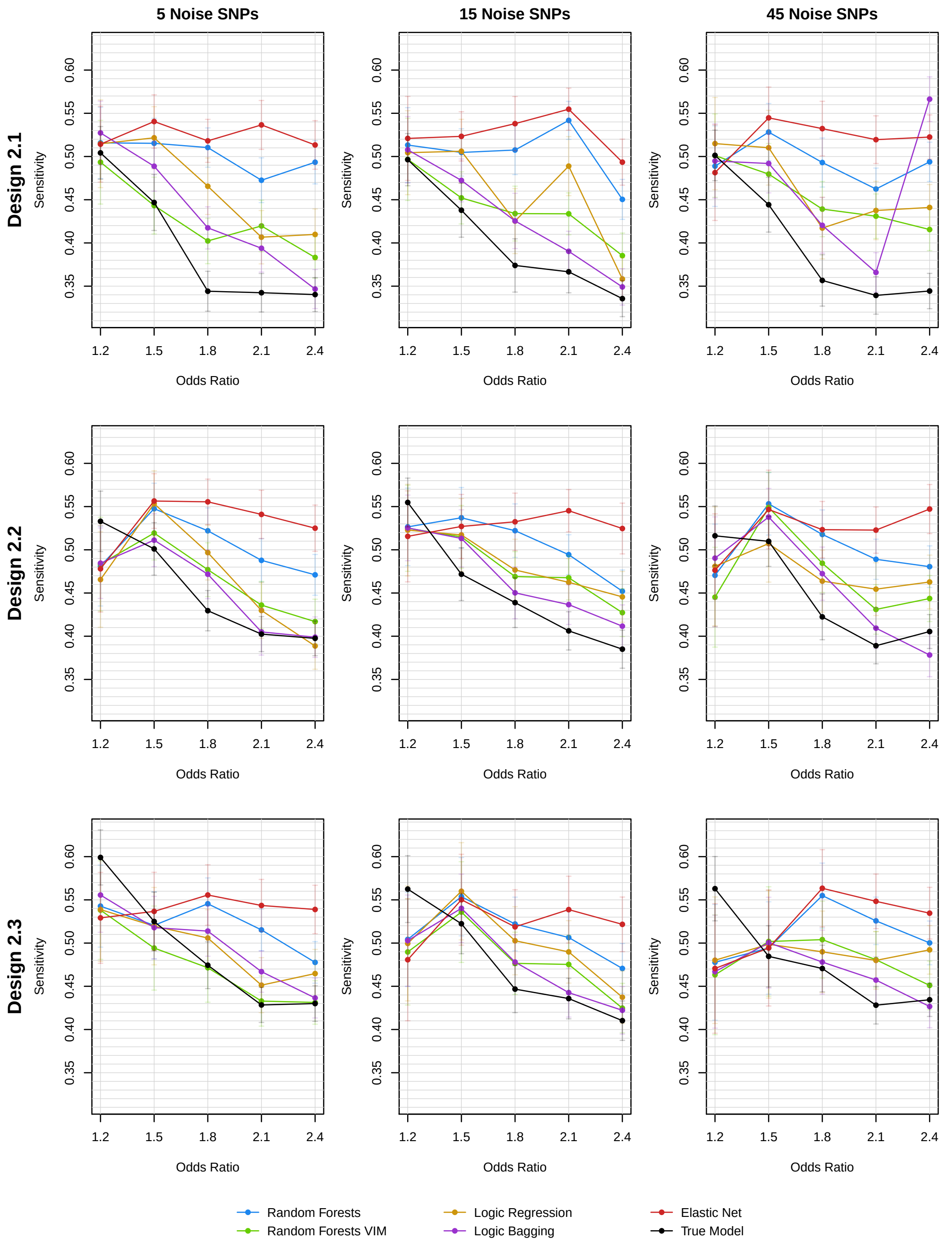

Figure S13: Mean sensitivity for random forests, random forests VIM, logic regression, logic bagging, elastic net, and the true underlying model in the second simulation scenario incorporating interactions of SNPs evaluated on the test data.

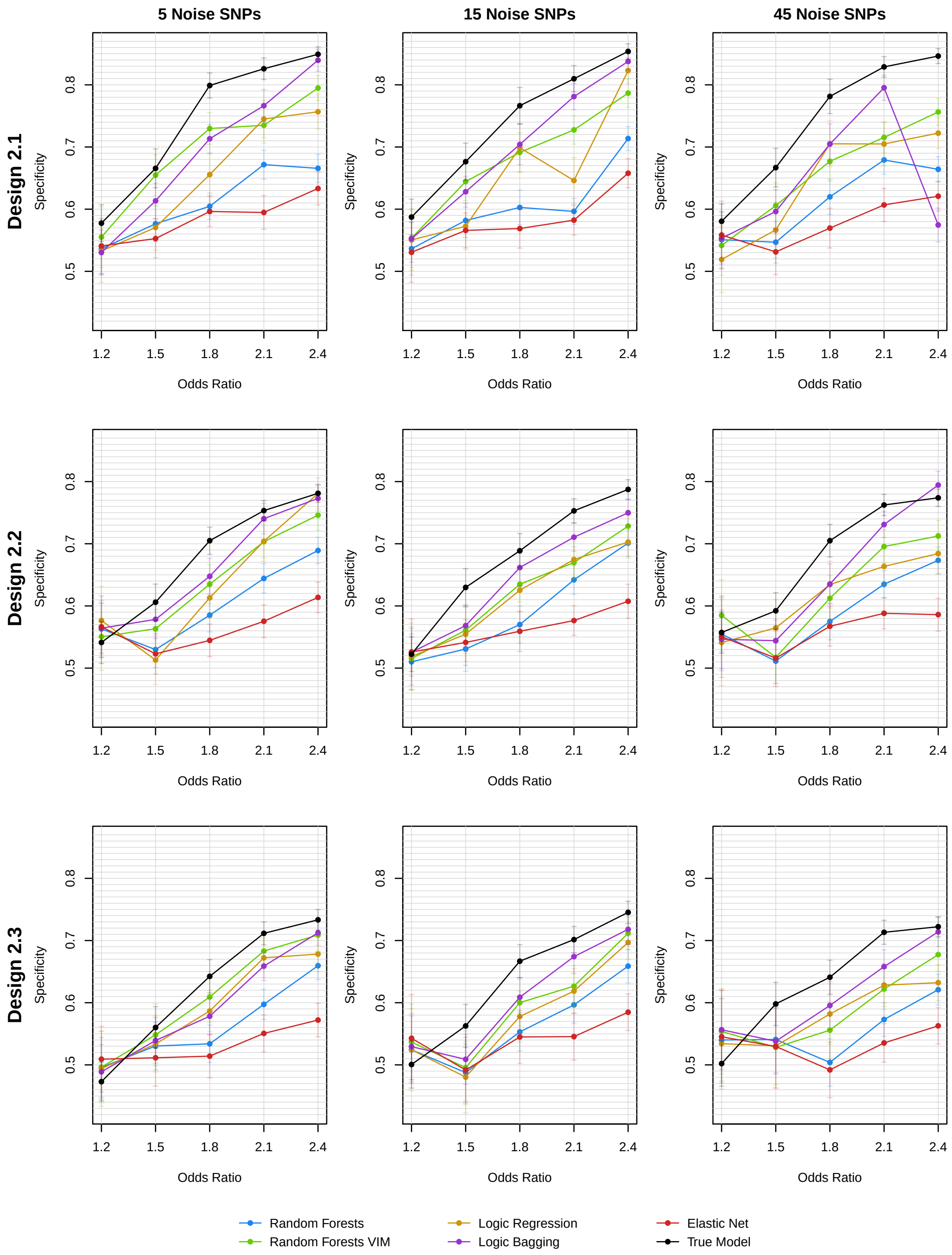

Figure S14: Mean specificity for random forests, random forests VIM, logic regression, logic bagging, elastic net, and the true underlying model in the second simulation scenario incorporating interactions of SNPs evaluated on the test data.

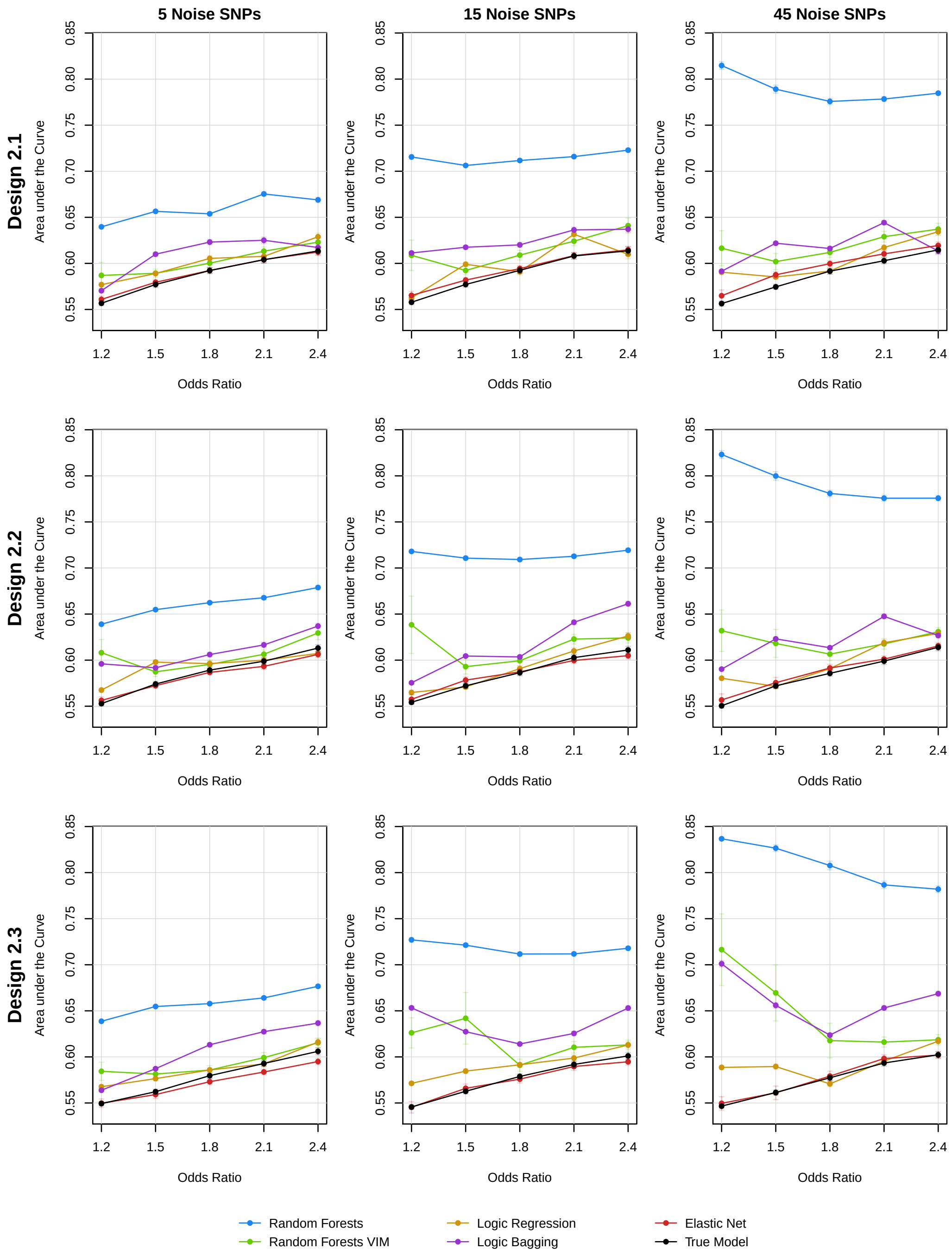

Figure S15: Mean AUC for random forests, random forests VIM, logic regression, logic bagging, elastic net, and the true underlying model in the second simulation scenario incorporating interactions of SNPs evaluated on the training data itself.

4.3 Gene-environment interactions

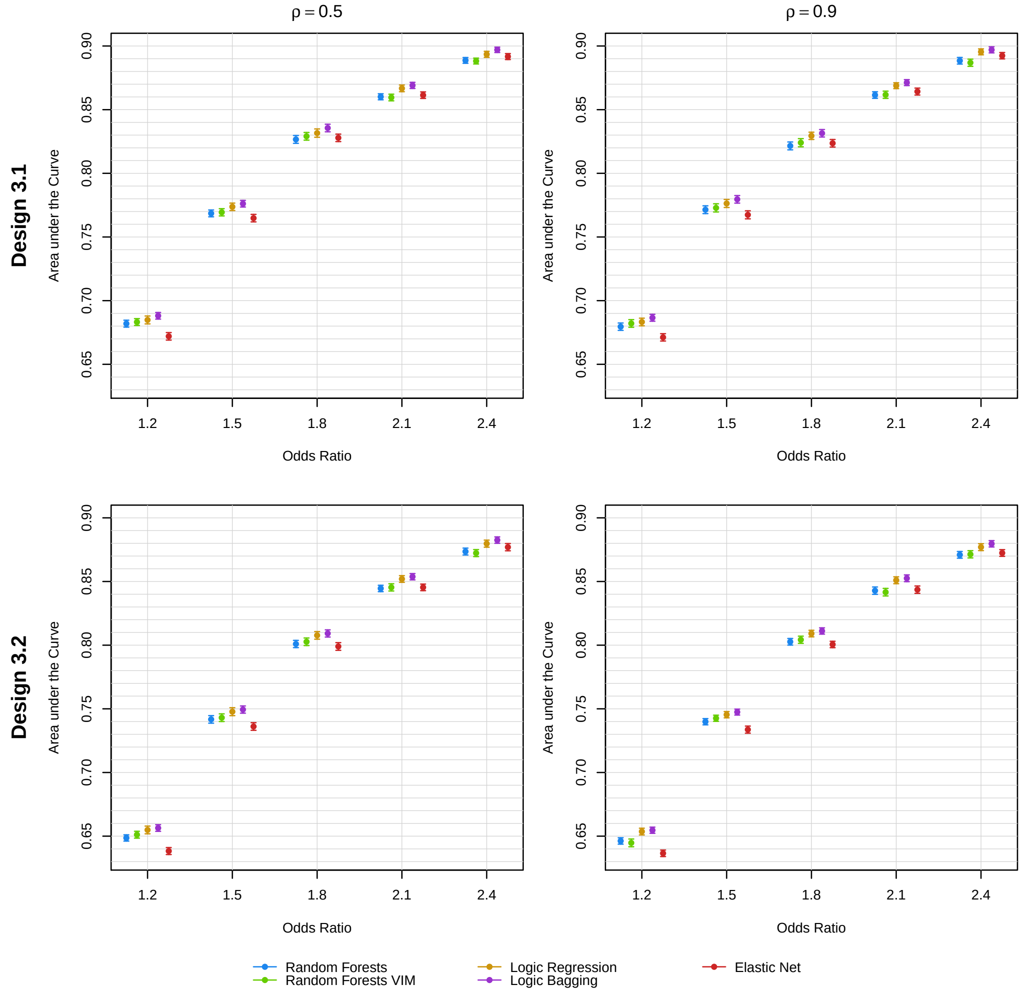

Figure S16: Mean AUC and asymptotic 95% confidence intervals for random forests, random forests VIM, logic regression, logic bagging, and elastic net in the third simulation scenario incorporating continuous input variables evaluated on the test data.

Table S1: Estimated type I error rate for random forests, random forests VIM, logic regression, logic bagging, and elastic net in the third simulation scenario incorporating continuous input variables evaluated on the test data.

Algorithm	Type I Error Rate
Random Forests	0.056
Random Forests VIM	0.052
Logic Regression	0.051
Logic Bagging	0.054
Elastic Net	0.020

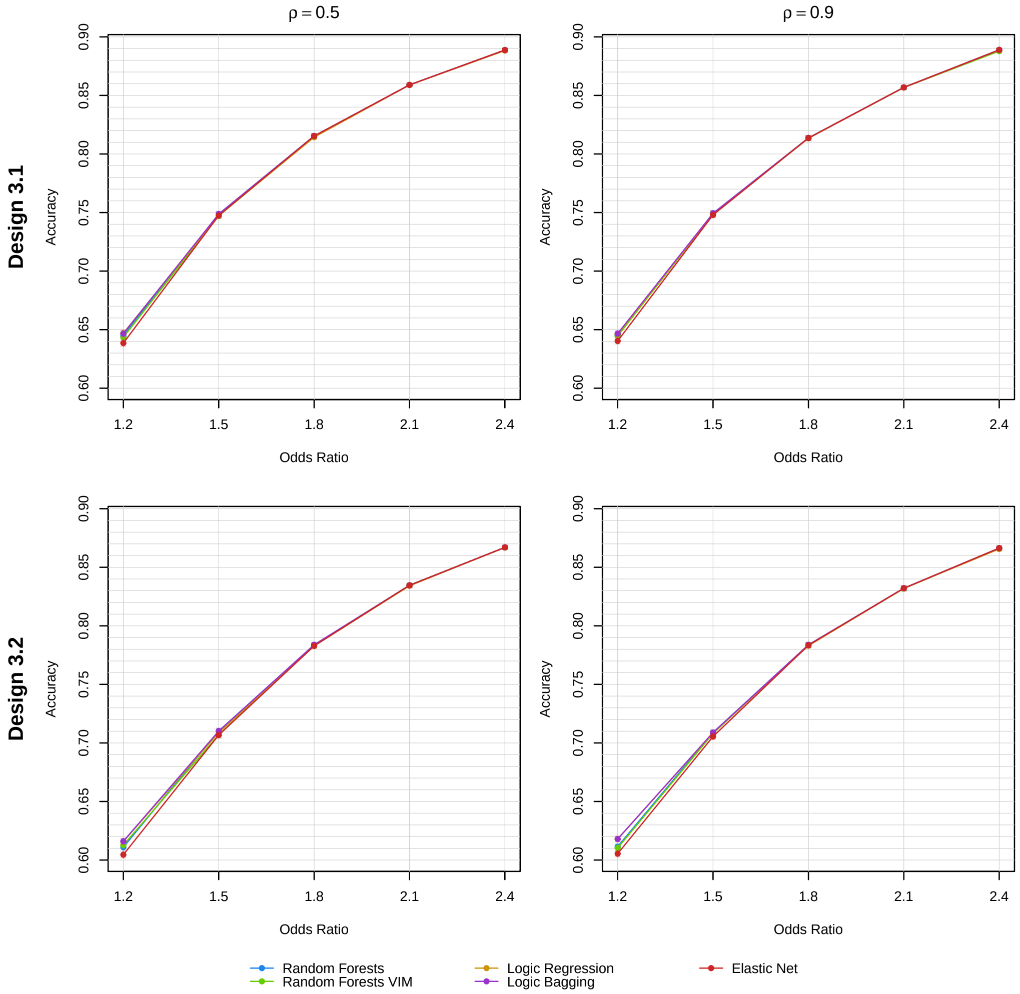

Figure S17: Mean accuracy for random forests, random forests VIM, logic regression, logic bagging, and elastic net in the third simulation scenario incorporating continuous input variables evaluated on the test data.

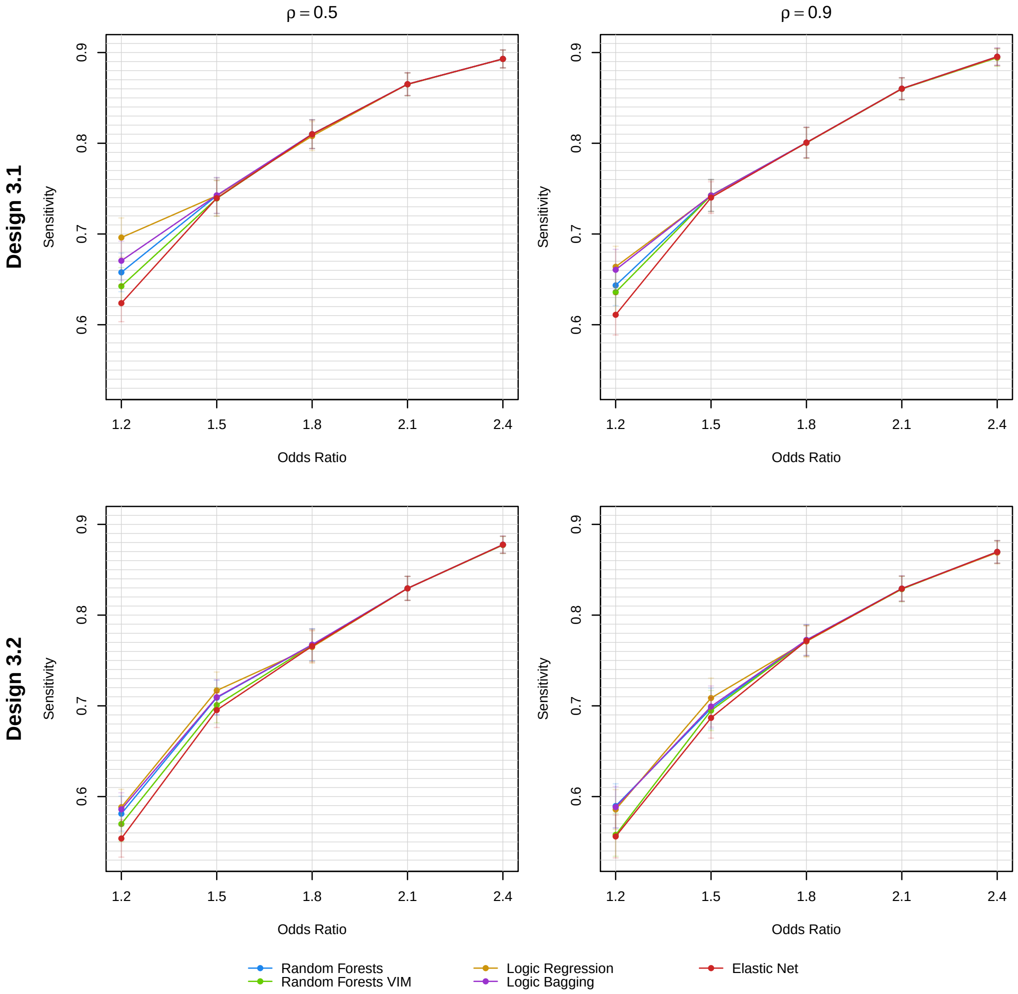

Figure S18: Mean sensitivity for random forests, random forests VIM, logic regression, logic bagging, and elastic net in the third simulation scenario incorporating continuous input variables evaluated on the test data.

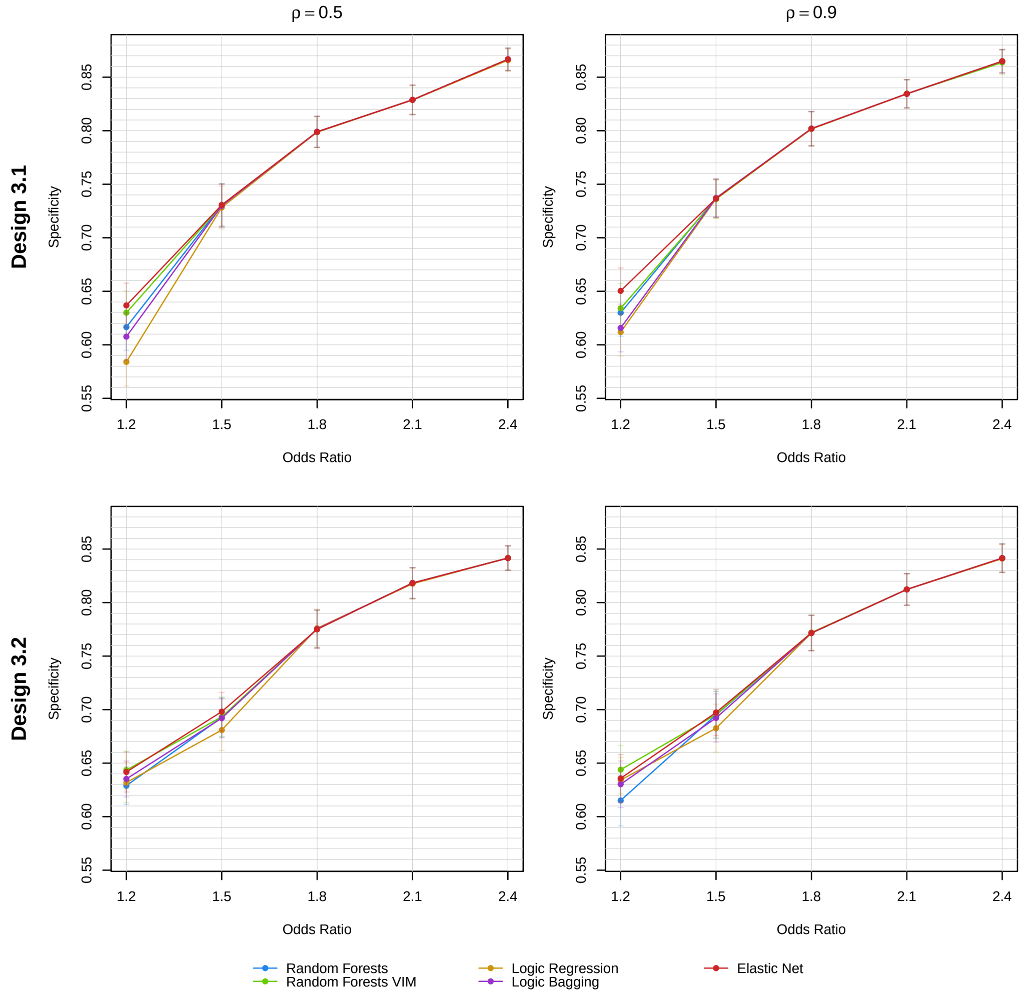

Figure S19: Mean specificity for random forests, random forests VIM, logic regression, logic bagging, and elastic net in the third simulation scenario incorporating continuous input variables evaluated on the test data.

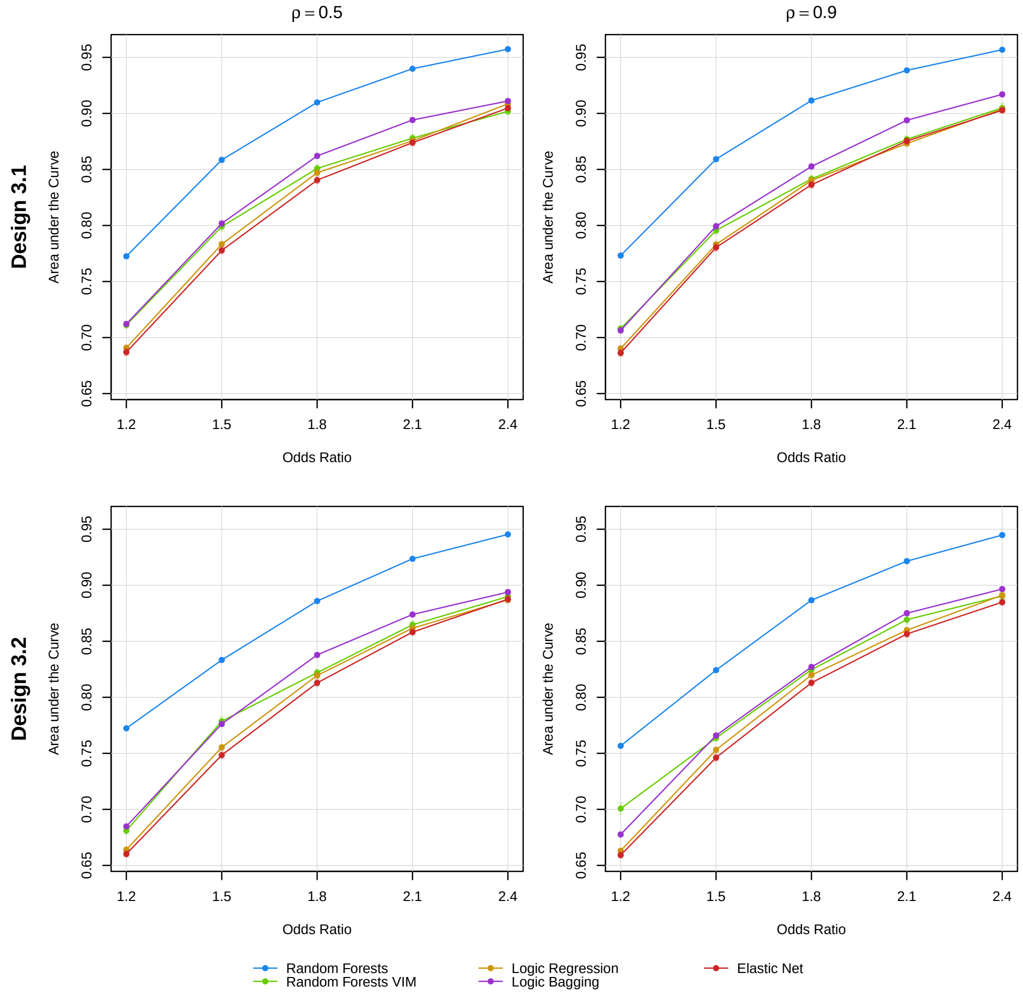

Figure S20: Mean AUC for random forests, random forests VIM, logic regression, logic bagging, and elastic net in the third simulation scenario incorporating continuous input variables evaluated on the training data itself.

4.4 Comparison considering binary SNP codings

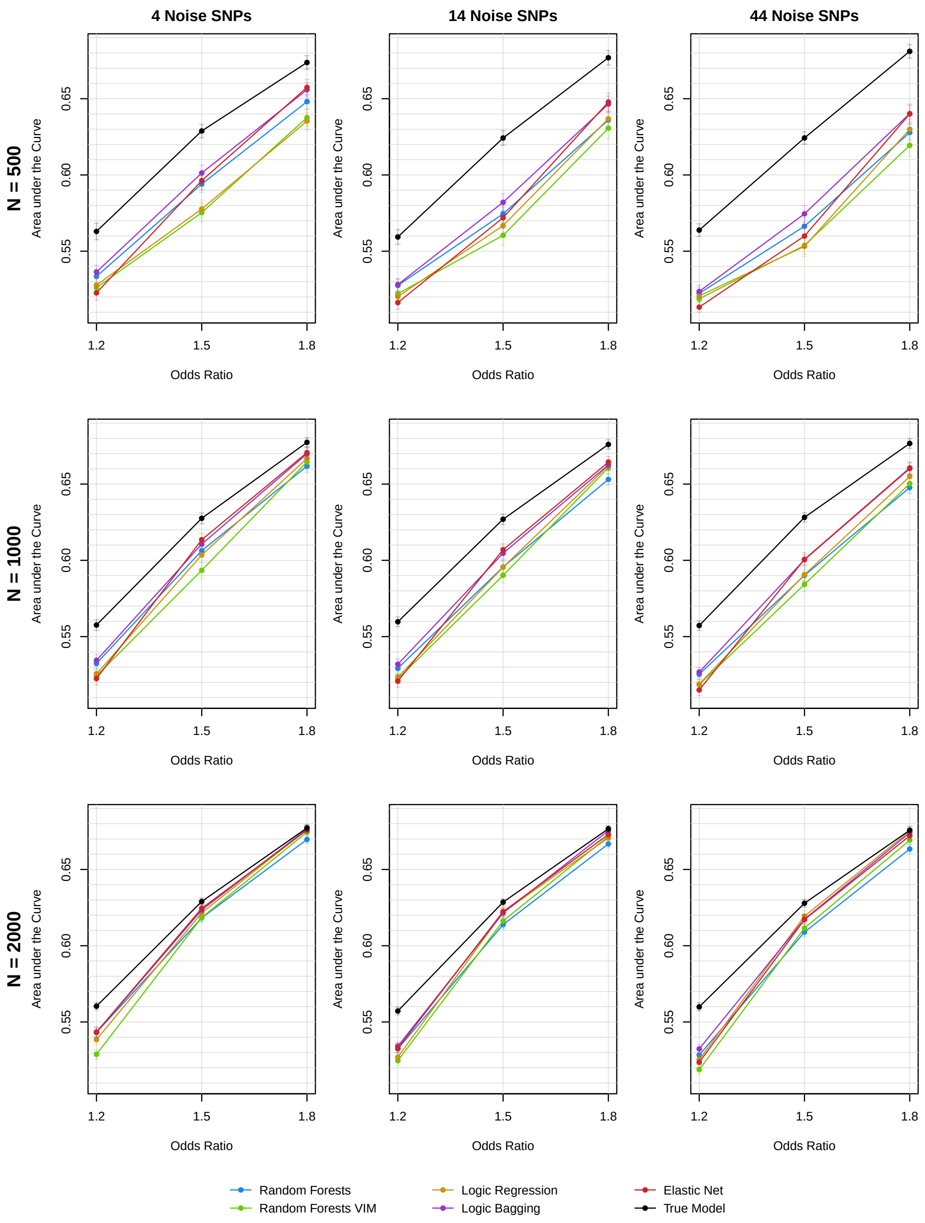

Figure S21: Mean AUC for random forests, random forests VIM, logic regression, logic bagging, elastic net, and the true underlying model in the first simulation scenario considering marginal effective SNPs evaluated on the test data. Here, the binary $\{0, 1\}$ SNP coding was used for each method.

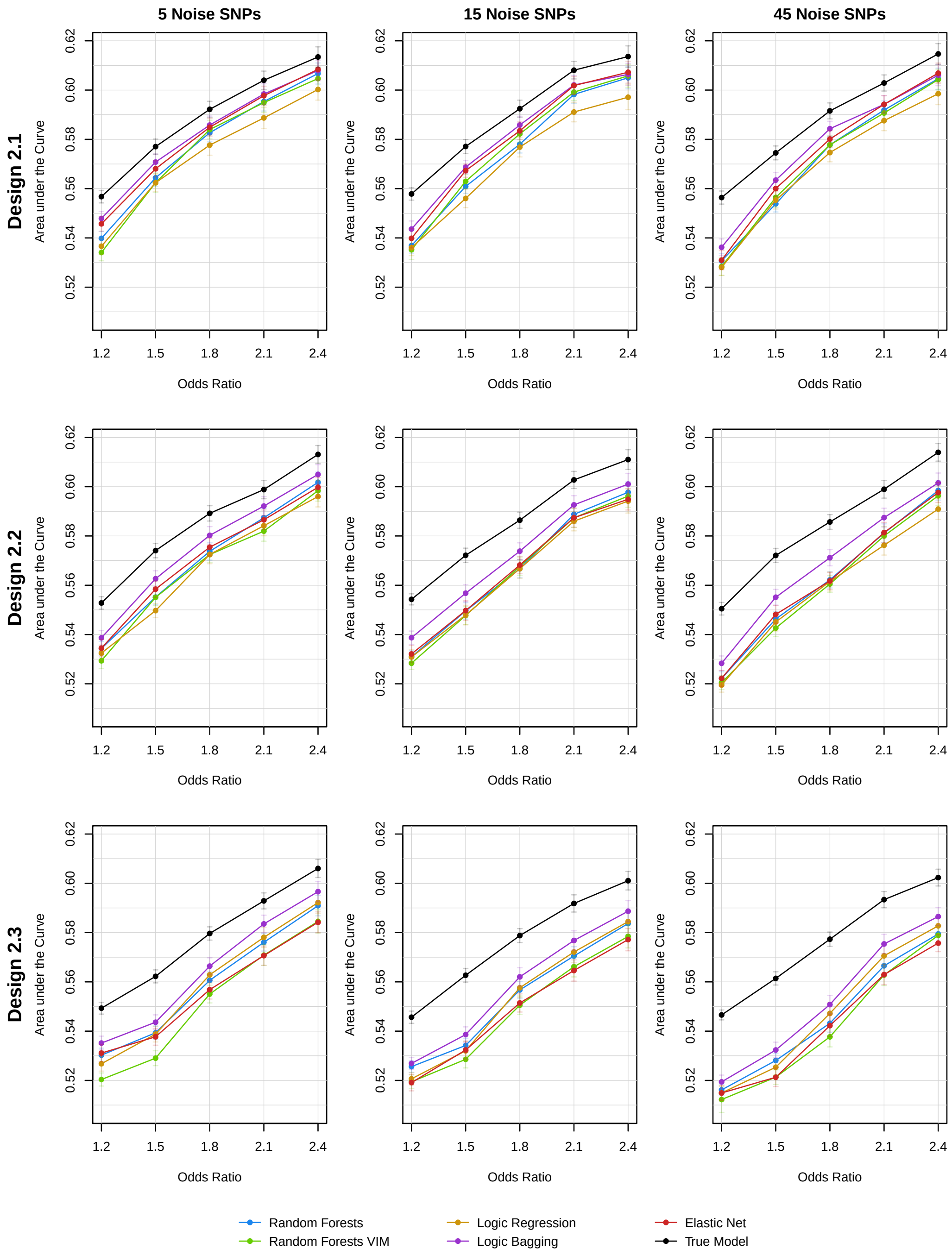

Figure S22: Mean AUC for random forests, random forests VIM, logic regression, logic bagging, elastic net, and the true underlying model in the second simulation scenario incorporating interactions of SNPs evaluated on the test data. Here, the binary $\{0, 1\}$ SNP coding was used for each method.

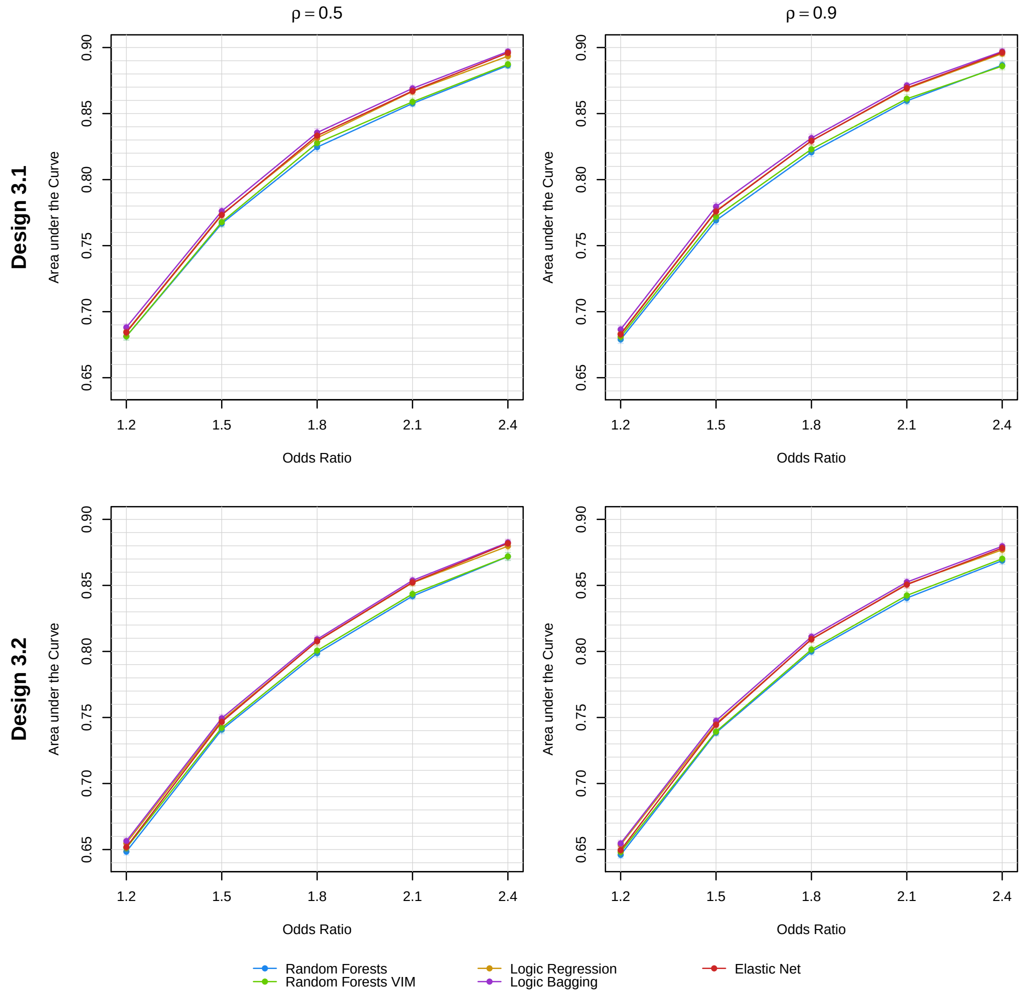

Figure S23: Mean AUC for random forests, random forests VIM, logic regression, logic bagging, and elastic net in the third simulation scenario incorporating continuous input variables evaluated on the test data. Here, the binary $\{0,1\}$ SNP coding was used for each method.

5 Real data application

Table S2: Median p-values of the Wald tests for the final age-adjusted models built on the SALIA data set

Term	Algorithm	NO_2	NO_x	PM_{10}	PM_{coarse}	$PM_{2.5}$	$PM_{2.5\ absorbance}$
GRS	Random Forests	0.469	0.385	0.531	0.550	0.332	0.539
	Random Forests VIM	0.485	0.432	0.416	0.470	0.404	0.449
	Logic Regression	0.430	0.420	0.394	0.452	0.338	0.400
	Logic Bagging	0.427	0.368	0.463	0.502	0.228	0.492
	Elastic Net	0.701	0.691	0.690	0.705	0.787	0.678
E	Random Forests	0.377	0.417	0.493	0.505	0.535	0.330
	Random Forests VIM	0.432	0.432	0.501	0.444	0.489	0.296
	Logic Regression	0.243	0.273	0.267	0.330	0.235	0.125
	Logic Bagging	0.378	0.388	0.485	0.539	0.513	0.249
	Elastic Net	0.304	0.356	0.425	0.333	0.421	0.250
GxE	Random Forests	0.489	0.538	0.575	0.591	0.511	0.530
	Random Forests VIM	0.505	0.402	0.401	0.460	0.457	0.490
	Logic Regression	0.467	0.404	0.417	0.432	0.440	0.407
	Logic Bagging	0.563	0.511	0.512	0.575	0.444	0.480
	Elastic Net	0.775	0.780	0.742	0.795	0.748	0.666

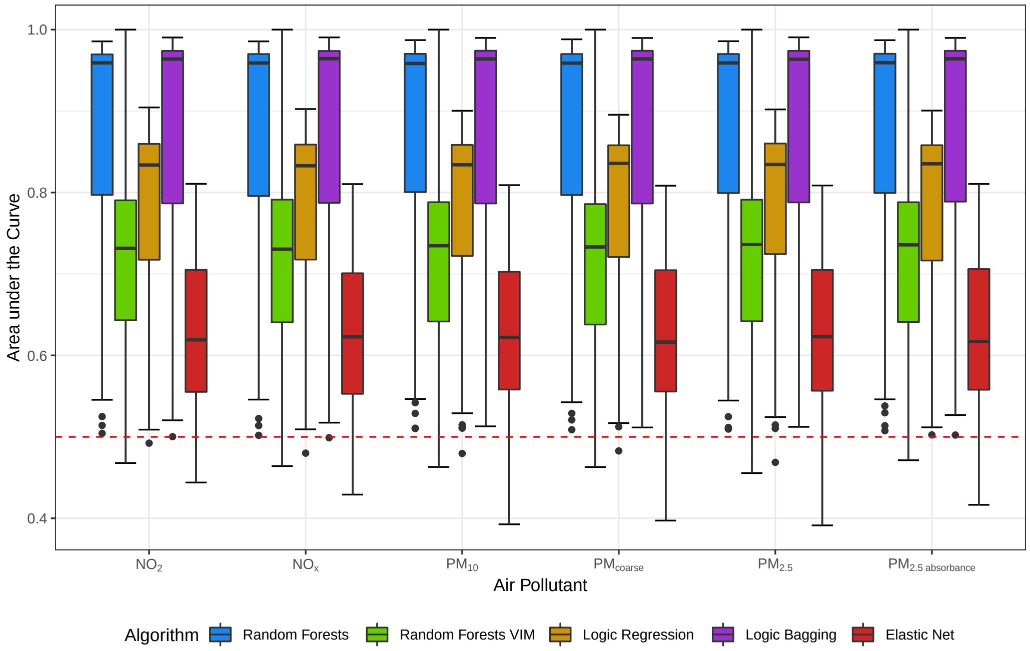

Figure S24: AUC for random forests, random forests VIM, logic regression, logic bagging, and elastic net in the application to data from the SALIA study evaluated on the training data itself. Results for the final age-adjusted models with different air pollution indicators.

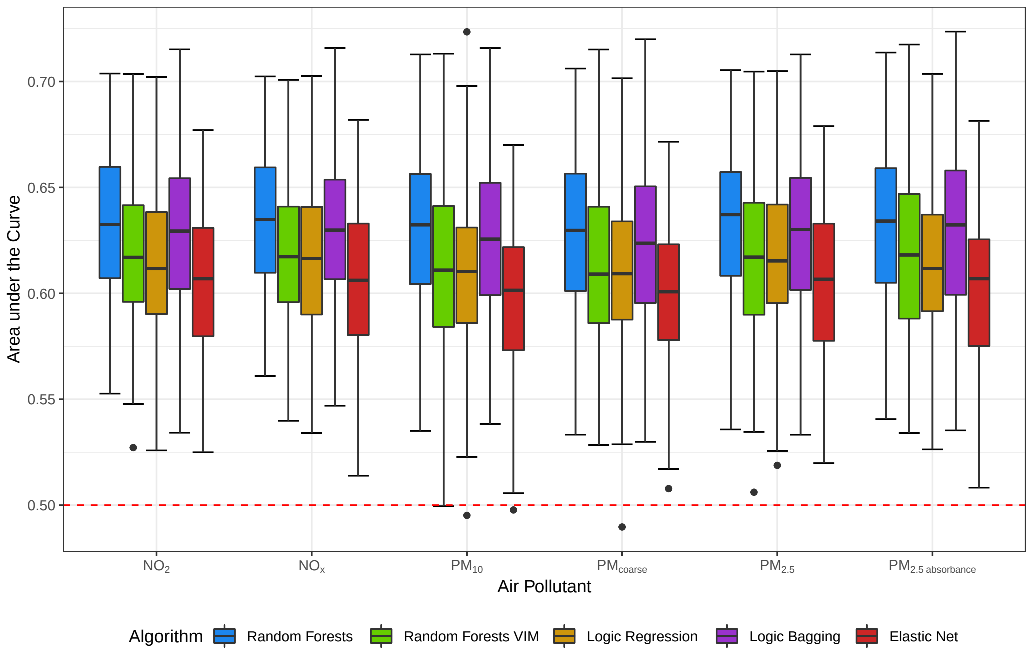

Figure S25: AUC for random forests, random forests VIM, logic regression, logic bagging, and elastic net in the application to data from the SALIA study evaluated on the test data. Results for the final age-adjusted models with different air pollution indicators. Current and former smokers were excluded from the base data set as part of a sensitivity analysis.

6 Distribution of the GRS

In the main effects simulation scenario and in the gene-gene interaction effect simulation scenario, the classification sensitivity is relatively low in some settings. This phenomenon can be explained by the need of dichotomizing the GRS into cases and controls for estimating the sensitivity and the discrete structure of the space of input variables/SNPs. To illustrate this, we present an exemplary GRS distribution occurring in the simulation study.

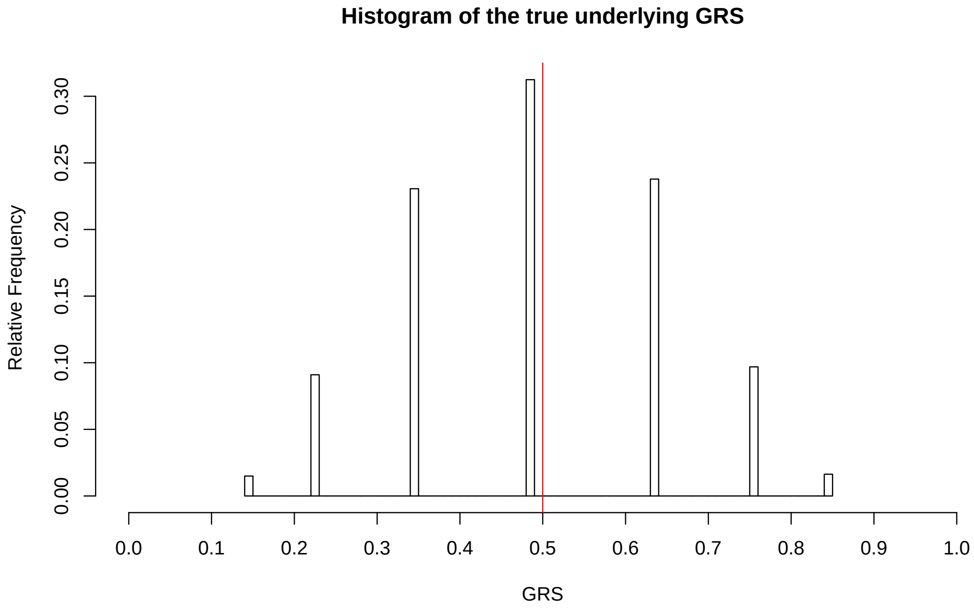

Figure S26: Histogram of the true underlying GRS for the main effects simulations scenario and the setting with an odds ratio of 1.8, 44 noise SNPs, and a sample size of $N = 2000$.
